# A new method for exploring gene–gene and gene–environment interactions in GWAS with tree ensemble methods and SHAP values

**DOI:** 10.1186/s12859-021-04041-7

**Published:** 2021-05-04

**Authors:** Pål V. Johnsen, Signe Riemer-Sørensen, Andrew Thomas DeWan, Megan E. Cahill, Mette Langaas

**Affiliations:** 1 SINTEF DIGITAL, Forskningsveien 1, 0373 Oslo, Norway; 2 Department of Mathematical Sciences, Norwegian University of Science and Technology, A. Getz vei 1, 7491 Trondheim, Norway; 3 Department of Chronic Disease Epidemiology and Center for Perinatal, Pediatric and Environmental Epidemiology, Yale School of Public Health, 1 Church Street, New Haven, CT 06510 USA; 4 Gemini Center for Sepsis Research, Department of Circulation and Medical Imaging, NTNU, Norwegian University of Science and Technology, Prinsesse Kristinas gate 3, 7030 Trondheim, Norway

**Keywords:** GWAS, Tree ensemble models, XGBoost, SHAP, Model explainability, Gene–gene and gene–environment interactions

## Abstract

**Background:**

The identification of gene–gene and gene–environment interactions in genome-wide association studies is challenging due to the unknown nature of the interactions and the overwhelmingly large number of possible combinations. Parametric regression models are suitable to look for prespecified interactions. Nonparametric models such as tree ensemble models, with the ability to detect any unspecified interaction, have previously been difficult to interpret. However, with the development of methods for model explainability, it is now possible to interpret tree ensemble models efficiently and with a strong theoretical basis.

**Results:**

We propose a tree ensemble- and SHAP-based method for identifying as well as interpreting potential gene–gene and gene–environment interactions on large-scale biobank data. A set of independent cross-validation runs are used to implicitly investigate the whole genome. We apply and evaluate the method using data from the UK Biobank with obesity as the phenotype. The results are in line with previous research on obesity as we identify top SNPs previously associated with obesity. We further demonstrate how to interpret and visualize interaction candidates.

**Conclusions:**

The new method identifies interaction candidates otherwise not detected with parametric regression models. However, further research is needed to evaluate the uncertainties of these candidates. The method can be applied to large-scale biobanks with high-dimensional data.

**Supplementary Information:**

The online version contains supplementary material available at 10.1186/s12859-021-04041-7.

## Background

In a traditional genome-wide association study (GWAS) each single nucleotide polymorphism (SNP) is tested individually for association with a particular phenotype. Using computationally efficient generalized or Bayesian linear mixed models that account for population stratification and cryptic relatedness, this approach can successfully identify risk alleles in the genome for complex diseases such as type 2 diabetes, Celiac disease and schizophrenia using large biobanks consisting of hundreds of thousands of individuals and SNPs [1–3]. Despite this, the estimated effects of the risk alleles are typically small and a large proportion of the estimated genetic heritability is yet to be explained for common traits and diseases [4]. One reason may be that most traits and diseases are highly polygenic, and thus many risk alleles with tiny effects will not be declared statistically significant due to stringent *p-*value significance thresholds. A second reason may be that the effect of the risk alleles are parametrically misspecified in the models. Model misspecification may lead to reduced power of detecting risk alleles [5, 6]. A third reason may be failure to account for *epistasis*, namely interactions between genes which together can impact the association with a certain phenotype [7, 8]. A fourth reason for the missing genetic heritability may be gene–environment interactions where the effect of a gene depends on some external environmental factor. Incorporating interactions in a generalized linear mixed model, particularly gene–gene interactions, remains a difficult task in GWAS due to the large number of interactions to investigate, the strict assumptions of the interaction effects needed and the multiple testing problem among other things [9].

In many situations the number of directly genotyped SNPs to evaluate, ignoring imputed genotype values, may be of the order of millions. With millions of SNPs to investigate the total number of SNP-pairs becomes of the order of $10^{12}$. For instance, with a family-wise error rate (FWER) less that 0.05, using the Bonferroni method this will require rejection of the null hypothesis of no interaction for *p*-values less than $10^{-14}$. Even with less conservative criteria than FWER, the small group of true interactions would be required to have very strong signals in order to be identified. Therefore, several two-stage algorithms have been developed such as GBOOST, SHEsisEpi and DSS where the first stage is a screening procedure to find the most promising gene–gene interactions, and the second stage is further investigation only based on these gene–gene interaction candidates [10–14]. However, inclusion of environmental features is either not considered or limited in the aforementioned two-stage algorithms [10, 15]. This can lead to overlooking important relationships including gene–environment interactions. Within modern biobanks, a rich amount of information, clinical, demographic, environmental and genetic, is available for each individual. A GWAS implemented using biobank data should therefore take full advantage of information with any perceived relevance for the trait of interest.

As an alternative to separately testing one parametric model for each interaction as well as the two-stage algorithms mentioned above, we suggest a nonparametric three-phase algorithm that can adjust for an unlimited number of features while searching for both gene–gene and gene–environment interaction candidates using tree ensemble models and SHAP values. We first rank the importance of each feature using the tree ensemble model XGBoost, a powerful prediction model suitable for high-dimensional data [16]. Recent research has demonstrated the possibility to interpret efficiently and with strong theoretical basis the importance of each feature from tree ensemble models using so-called SHapley Additive exPlanation (SHAP) values [17]. Based on this ranking, we further propose a model fitting process where the aim is to find the best XGBoost models with respect to predictive performance. The idea is that better predictive performance is a result of revealing additional relationships. Finally, based on these models, the aim is to explain the relationships that the models consider most important, and specifically the interactions. This type of procedure is more inclusive in order to find true interactions with the intention that these interaction candidates will need to be thoroughly investigated in a second stage. By using real data from UK Biobank, we demonstrate these models’ capability to: (a) Rank features by importance and thereby removing noise. (b) Evaluate the use of XGBoost as both a predictive model and explainable model, and finally (c) Rank and explain plausible gene–gene and gene–environment interactions. We finish by comparing the top ranked interactions with logistic regression with interaction terms and perform statistical tests. We will in addition do a stratified analysis of the interaction candidates. In this paper, the focus is on a case-control setting, but the method outlined in this paper can be applied to both continuous and discrete phenotypes. Obesity was selected since this particular trait has been extensively researched in previous GWAS [18–20] providing a meaningful way to evaluate our method.

## Methods

Recent research within GWAS to account for both genetic and environmental interactions have focused on how to explore the large amount of data in a more systematic way by using various nonparametric machine learning models such as tree ensemble models and deep neural networks [21–23]. So far, the most successfully applied machine learning methods for genotype data are tree ensemble models such as gradient tree boosting models [24] first introduced by Jerome H. Friedman [25], but with subsequent improvements. One such improvement is the so-called XGBoost implementation [16] used in this paper. XGBoost, as any tree ensemble model, consists of many so-called *weak learners* which in our case are *regression trees*. There are several advantages of using trees as they can naturally handle data of mixed type (continuous, categorical etc.) and missing values, they have the ability to deal with irrelevant and correlated variables, and they are computationally efficient to use [26]. However, trees suffer from low predictive power, high variance, lack of smoothness, and inability to capture linear structures. High variance and overfitting are of greater concern with deeper trees. Tree ensemble models, consisting of many trees, will reduce this variance and improve the predictive power [26]. Smoothness and ability to capture linear structures have also been shown to be improved [27]. The concern about using tree ensemble models within GWAS has been how to objectively evaluate the importance of each feature similar to *p*-values in traditional GWAS. However, a recent paper by Lundeberg et al. [17] showed that tree ensemble models have the capability to be efficiently and objectively interpreted by measuring the importance of each feature with respect to the predictions of the model by introducing so-called SHAP values. Interpretation of the XGBoost models through SHAP values will allow us to explain the prediction for each individual, a beneficial property in a precision medicine setting.

### Problem description and syntax

Let $y_i$ be the value/phenotype of some trait for individual *i*. This value may signify the absence or presence of a certain trait, such as a disease, or some continuous measure such as height, weight or blood pressure, or even a combination of measures such as the body mass index (BMI). Let $g_{i,a}$ denote the number of copies (0, 1 or 2) of the minor allele (referred to as the genotype) for a biallelic SNP *a* and individual *i*. Furthermore, let $x_{i,e}$ denote the value of some environmental feature, and let the matrix $\mathbf{X }_{N \times M}$ represent all genetic and environmental data for all *N* individuals and *M* features. Usually in a GWAS, the association between a SNP and a trait is tested separately for each SNP. However, another approach is to model the association between several SNPs and a trait simultaneously. We will use the latter approach, and will refer to genetic and environmental data as *features*, $\mathbf{x }_i$, for each individual *i*. Consider a model for predicting the phenotype, $y_i$, denoted ${\hat{y}}_i(\mathbf{x }_i)$. The performance of the model depends on how close each ${\hat{y}}_i(\mathbf{x }_i)$ is to $y_i$ for all individuals with respect to some loss function. However, equally important in this setting is to understand what influences the prediction ${\hat{y}}_i(\mathbf{x }_i)$. In other words, we would like to understand how *each feature* contributes to the prediction ${\hat{y}}_i(\mathbf{x }_i)$ for each individual *i*. In this paper we aim to derive such a model and we will specifically consider the special case where the trait $y_i$ is binary, that is, presence or absence of a phenotype. We denote the group consisting of individuals where the phenotype is absent as the *control group*, and the other group as the *case group*.

Before introducing our tree ensemble- and SHAP-based method for identifying interaction candidates, we will outline the necessary building blocks applied in our method including the choice of tree ensemble model, the performance metric to use in a binary classification setting as well as which metrics to use in order to evaluate the importance of each feature.

### XGBoost

**Fig. 1:**
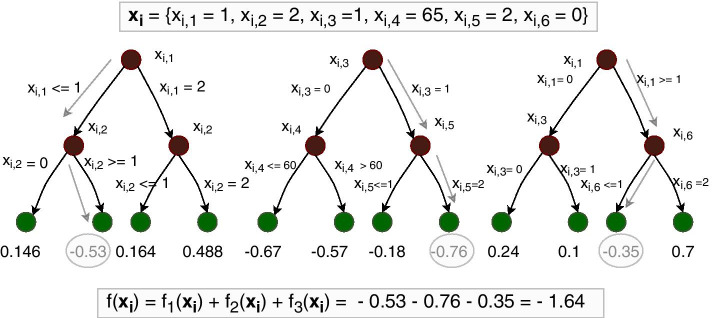
An example with three constructed regression trees with six features $x_{i,1}$ to $x_{i,6}$ used as splitting points at each branch, and leaf node values. Also shown is the computation of $f({\mathbf {x}}_i)$ given an example of feature values ${\mathbf {x}}_i$. The structure of the trees opens the possibility to explore interactions since a path from a root node to a leaf node denotes a combination of feature values

The XGBoost tree ensemble model consists of several regression trees, as illustrated in Fig. 1. An important aspect of trees, is that they automatically handle interactions between features. Consider the leftmost tree in Fig. 1, where the first split is for feature $x_1$, and then for both branches of the tree the next split is for feature $x_2$. Observe that the impact of feature $x_2$ in the tree is dependent on the value of feature $x_1$, with a different outcome if $x_1\le 1$ than if $x_1=2$. This means that a statistical interaction between feature $x_1$ and $x_2$ is encoded in the tree.

#### Constructing trees

The XGBoost algorithm starts with the construction of a single regression tree, and then new regression trees are consecutively constructed in a gradient boosting matter based on a loss function. The loss function is a sum of a loss function per individual, $\ell (y_i,{\hat{y}}^{(T)}_i(\mathbf{x }_i))$, which is a differentiable convex function. It measures the performance of the prediction, ${\hat{y}}^{(T)}_i(\mathbf{x }_i)$, with respect to the observed response, $y_i$, for observation *i* with features $\mathbf{x }_i$ when there is a total of *T* trees in the model. In a binary classification setting a convenient loss function is the binary cross-entropy:

$$\begin{aligned} \ell (y_i,{\hat{y}}^{(T)}_i(\mathbf{x }_i)) = -y_i \log ({\hat{y}}^{(T)}_i(\mathbf{x }_i)) - (1-y_i) \log (1-{\hat{y}}^{(T)}_i(\mathbf{x }_i)). \end{aligned}$$

Regression tree number $\tau$ is denoted as $f_{\tau }$, a data structure that contains information of nodes, features used as splitting points and leaf node values. The function $f_{\tau }(\mathbf{x }_i)\in {\mathbb {R}}$ outputs the value of the leaf node (green circles in Fig. 1) corresponding to features ${\mathbf {x}}_i$ based on tree $\tau$. In a binary classification setting, the prediction ${\hat{y}}^{(T)}_i({\mathbf {x}}_i)$ is interpreted as the probability that individual *i* is a case given a total of *T* regression trees.

In order for ${\hat{y}}^{(T)}_i(\mathbf{x }_i)$ to represent a probability, a much used transformation is the sigmoid function:

1 $$\begin{aligned} {\hat{y}}^{(T)}_i(\mathbf{x }_i) = \frac{1}{1 + e^{-\sum _{\tau =1}^{T} f_{\tau }(\mathbf{x }_i)}}. \end{aligned}$$

When constructing each tree, one starts at the root node and successively investigates which feature to use as a splitting point at each node. The model will choose the split that minimizes the total loss function at that point. There are different strategies when constructing the trees. Splitting at the node which gives the largest decrease in loss is the approach that will be used in our case. The XGBoost R software package applies the histogram method to reduce the search time [28–30]. For the handling of missing values, we refer to the original XGBoost paper [16].

The model will typically stop training when the total loss function has not decreased in a given number of iterations, where a new regression tree is constructed in each iteration. The prediction of the final model on the logit scale given features $\mathbf{x }_i$ is given by $f(\mathbf{x }_i) = \sum _{\tau = 1}^T f_{\tau } (\mathbf{x }_i)$, while the probability of the case class will be calculated using the sigmoid transform on $f(\mathbf{x }_i)$, as in Eq. (1).

#### Hyperparameters in XGBoost

XGBoost has a large set of hyperparameters, which may influence the performance of the algorithm and its ability to find the best representation of the data. In this paper, we focus on the learning rate $\eta$, *subsample*, *colsample_bytree*, *colsample_bylevel* and *max_depth*. The learning rate $\eta \in (0,1]$ scales the values of the leaf nodes after the construction of each new tree, in which case $f_t(\mathbf{x }_i) = \eta f^{*}_t(\mathbf{x }_i)$ where $f^{*}_t(\mathbf{x }_i)$ is the raw regression tree before the scaling of the leaf node values has been applied. This will limit particular trees to dominate the prediction. It has been shown to be important since it influences how fast the model will learn and it can prevent early overfitting. In high-dimensional problems this is crucial and the learning rate should be well below 1 and is typically 0.1 or smaller [26, 31]. The subsample and colsample_bytree hyperparameters decide the proportion of individuals and features to be evaluated in each regression tree respectively. They also prevent overfitting, and in addition reduce the training time of the model. A typical value for both hyperparameters is 0.5, and in high-dimensional data it has been proposed that even smaller values can be beneficial [26]. However, this will depend on what proportion of the high-dimensional data is relevant. If the relevant proportion is small, a more reasonable value is closer to 1 [16]. The parameter colsample_bylevel is used to partition the number of possible features to use as splitting points in each level of the tree. The literature is quite scarce on its effect, but it may oppose the non-optimal greedy approach search as well as providing more room for learning in a way similar to the learning rate. The parameter max_depth is the maximum depth in each tree. Other important hyperparameters are the regularization parameter $\lambda$ described in Chen and Guestrin [16] as well as the parameter early_stopping_rounds which is the maximum number of rounds without predictive improvement of the validation data before the training stops. To avoid overfitting, the validation data is independent of the training data.

### Classification performance metric

For a binary classification model, the predictive performance in the validation data can be evaluated with specific focus on the group that is of particular interest (the case group). Let TP, FP and FN be the number of true positives, false positives and false negatives, respectively. The precision and recall given the classifications from a model are defined as follows,

$$\begin{aligned} {{\,\mathrm{Precision}\,}}= & {} \frac{{{\,\mathrm{TP}\,}}}{{{\,\mathrm{TP}\,}}+{{\,\mathrm{FP}\,}}}, \\ {{\,\mathrm{Recall}\,}}= & {} \frac{{{\,\mathrm{TP}\,}}}{{{\,\mathrm{TP}\,}}+ {{\,\mathrm{FN}\,}}}. \end{aligned}$$

A convenient measure for the model performance is the area under the curve, denoted PR-AUC (precision-recall area-under-curve) [32]. PR-AUC is most often used in the case of imbalance, meaning that one group is larger than the other. When TP = 0 and FP = 0, corresponding to a model that always predicts an individual to be a control, the precision is defined to be zero.

### A measure of feature importance in tree ensemble models - SHapley Additive exPlanation (SHAP) values

When evaluating the global feature importance in a tree ensemble model, one possibility is to look at the relative decrease in loss for all splits by a given feature over all trees [33]. Unfortunately, this measure suffers from so-called *inconsistency* as discussed in Lundberg et al. [34]. In short, this means that the feature contributions are unfairly distributed as a result of not accounting for the importance of the order in which the features are introduced in the trees. Another popular, but similarly inconsistent, importance metric is counting the number of times each feature is used as a splitting point. Instead, a metric based on so-called SHapley Additive exPlanation (SHAP) values can be shown to achieve consistency [17, 35]. In the case of tree ensemble models, each feature *j* for each individual *i* is given a SHAP value, $\phi _{i,j}$, which represents the contribution of feature *j* with respect to the prediction, $f(\mathbf{x }_i) = \sum _{\tau =1}^{T} \eta f^{*}_{\tau }(\mathbf{x }_i)$, equal to the output of the linear sum of all *T* regression trees in a tree ensemble model given features $\mathbf{x }_i$. This metric exhibits several favourable properties aside from consistency [35]. For instance, the sum of the contributions of each feature, $\phi _{i,j}$, including a constant $\phi _0$ equals the prediction of the model $f(\mathbf{x }_i)$:

2 $$\begin{aligned} f(\mathbf{x }_i) = \phi _0 + \sum _{j=1}^M \phi _{i,j}, \end{aligned}$$

where *M* is the number of features included in the model. Moreover, the total contribution of a subset of all features for each individual is simply equal to the sum of the SHAP values for each feature. The reason for these favourable properties is that the contribution, $\phi _{i,j}$, is computed based on a concept from game theory first introduced by Lloyd Shapley [36]:

3 $$\begin{aligned} \phi _{i,j} = \sum _{{\mathcal {S}} \subseteq {\mathcal {M}} \setminus \{j\} } \frac{|{\mathcal {S}}|!(M-|{\mathcal {S}}|-1)!}{M!}\left[ v_i({\mathcal {S}} \cup j)-v_i({\mathcal {S}})\right] , \end{aligned}$$

where ${\mathcal {M}}$ is the set of all features included in the model, the function $v_i({\mathcal {S}})$ measures the total contribution of a given set of features $(v_i({\mathcal {M}}) = f(\mathbf{x }_i)$), and the sum is across all possible subsets where feature *j* is not included. The parameter $\phi _0$ is defined as $\phi _0 = v({\mathcal {S}}=\emptyset )$. The key idea is that the contribution of each feature for each individual is measured by evaluating the difference between the prediction when the value of feature *j* is known, versus the case when the value feature *j* is unknown for all subsets ${\mathcal {S}} \subseteq {\mathcal {M}} \setminus \{ j \}$. In a statistical setting, the *marginal expectation* first introduced in Janzing, Minorics, and Blöbaum [37] seems to be a reasonable measure:

$$\begin{aligned} v_i({\mathcal {S}} \cup j) = E[f(\mathbf{X }_{i,{\mathcal {S}} \cup \{j\}}=\mathbf{x }^{*}_{i,{\mathcal {S}} \cup \{j\}},\mathbf{X }_{i,\overline{{\mathcal {S}} \cup \{j\}}})] \end{aligned}$$

where $E[f(\mathbf{X }_{i,{\mathcal {S}} \cup \{j\}}=\mathbf{x }^{*}_{i,{\mathcal {S}} \cup \{j\}},\mathbf{X }_{i,\overline{{\mathcal {S}} \cup \{j\}}})]$ is the expected prediction when only the values of the feature subset ${\mathcal {S}}$ as well as feature *j*, denoted $\mathbf{x }^{*}_{i,{\mathcal {S}} \cup \{j\}}$, are known, while the vector of the complement set, $\mathbf{X }_{i,\overline{{\mathcal {S}} \cup \{j\}}}$, is regarded as a random vector. Notice that ${\mathcal {S}} \cup \overline{{\mathcal {S}}} = {\mathcal {M}}$. The values $\phi _{i,j}$ in Expression (3) with $v_i({\mathcal {S}})$ measured as marginal expectations are denoted as SHAP values [35]. In the case of binary classification using a tree ensemble model, the prediction $f(\mathbf{x }_i)$ can be interpreted as the log-odds prediction.

By assuming all features are mutually independent, Lundberg et al. [17] constructed an algorithm to estimate the SHAP values in polynomial running time, $O(TLD^2)$, with maximum depth *D* and maximum number of leaves *L* in all *T* trees. The assumption about mutual independence is a limitation, and without this assumption the estimation of the SHAP values becomes more complicated [38]. For further details about estimations of SHAP values assuming mutual independence, see Additional File 1.

#### SHAP interaction value

The SHAP values can be further generalized to interpret pairwise interactions through the SHAP interaction values $\Phi _{i,j,k}$, $j \ne k$, for individual *i* and features *j* and *k* given by [17, 39]:

4 $$\begin{aligned} \Phi _{i,j,k} = \sum _{{\mathcal {S}} \subseteq {\mathcal {M}} \setminus \{j,k\}} \frac{|{\mathcal {S}}|!(M-|{\mathcal {S}}|-2)!}{2(M-1)!}\nabla _{i,j,k}({\mathcal {S}}), \end{aligned}$$

where

$$\begin{aligned} \begin{aligned} \nabla _{i,j,k}({\mathcal {S}})&= \left[ E[f(\mathbf{X }_{i,{\mathcal {S}} \cup \{j,k\}}=\mathbf{x }^{*}_{i,{\mathcal {S}} \cup \{j,k\}},\mathbf{X }_{i,\overline{{\mathcal {S}} \cup \{j,k\}}})]\right. \\&\quad \left. -E[f(\mathbf{X }_{i,{\mathcal {S}} \cup \{k\}}=\mathbf{x }^{*}_{i,{\mathcal {S}} \cup \{k\}},\mathbf{X }_{i,\overline{{\mathcal {S}} \cup \{k\}}})]\right] \\&\quad - \left[ E[f(\mathbf{X }_{i,{\mathcal {S}} \cup \{j\}}=\mathbf{x }^{*}_{i,{\mathcal {S}} \cup \{j\}},\mathbf{X }_{i,\overline{{\mathcal {S}} \cup \{j\}}})] -E[f(\mathbf{X }_{i,{\mathcal {S}}}=\mathbf{x }^{*}_{i,{\mathcal {S}}},\mathbf{X }_{i,\overline{{\mathcal {S}}}})]\right] . \end{aligned} \end{aligned}$$

If feature *k* yields additional information when present simultaneously with feature *j*, $\nabla _{i,j,k}({\mathcal {S}})$ will be different from zero with the sign depending on how feature *k* (when present) affects feature *j*. With these definitions, the pairwise SHAP interaction values have the same properties as the single-feature SHAP values. For instance, the contribution of a given feature *j*, $\phi _{i,j}$, can be separated into the contribution of *j* itself, denoted $\Phi _{i,j,j}$, in addition to all interactions including feature *j*, denoted as $\Phi _{i,j,k}$, for all $k \ne j$:

$$\begin{aligned} \phi _{i,j} = \Phi _{i,j,j} + \sum _{j \ne k} \Phi _{i,j,k}. \end{aligned}$$

The final prediction for each individual can be decomposed into

5 $$\begin{aligned} f(\mathbf{x }_i) = \phi _0 + \sum _{j=1}^M \phi _{i,j} = \phi _0 + \sum _{j=1}^M \left[ \Phi _{i,j,j} + \sum _{k \ne j} \Phi _{i,j,k} \right] , \end{aligned}$$

where $$\Phi _{i,j,k} = \Phi _{i,k,j}$$.

The interactions for all possible pairs of features for a particular tree ensemble model can be computed in $O(TMLD^2)$ time [17].

### Tree ensemble- and SHAP-based method for identifying interaction candidates

**Fig. 2:**
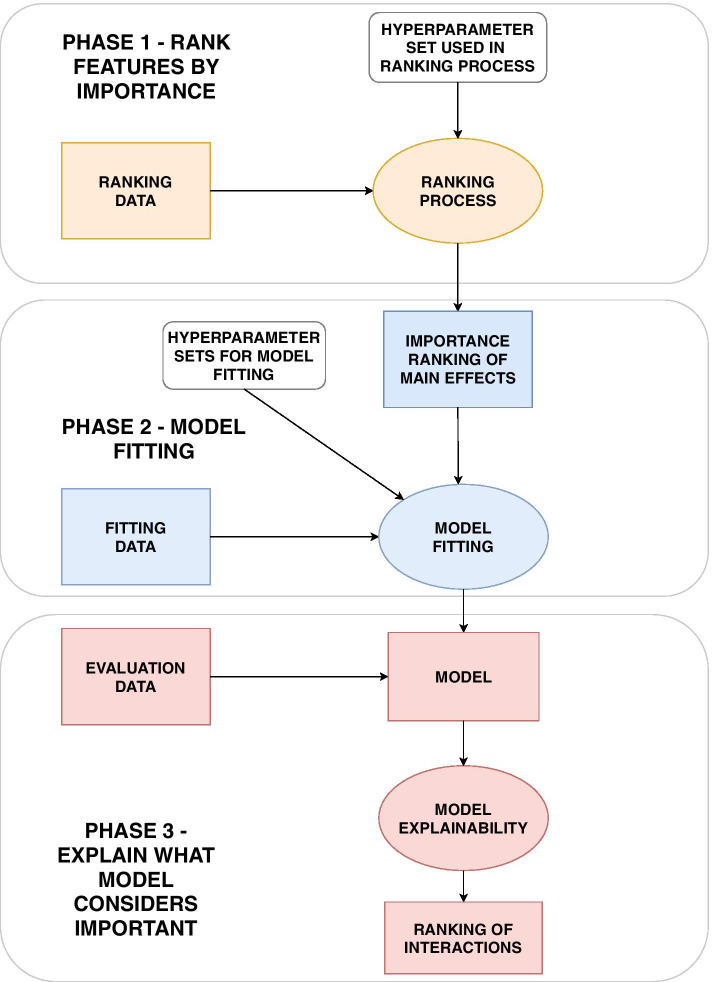
The ranking, model fitting and explanation phases. In the ranking phase, the SNPs and environmental features are ordered by their relative importance. The ranking is achieved with XGBoost and SHAP values as explained in Fig. 4. In the model fitting process, the top ranked features are combined and modelled with XGBoost as described in Fig. 6. Finally, the explanations and interactions are obtained from the SHAP values. This is visualized in Figs. 10, and 11

We propose a new method using XGBoost and SHAP values to identify potential interactions such as SNP-SNP interactions or SNP-environment interactions, but also nonparametric single-SNP effects. The method is outlined in Fig. 2.

We use a tree ensemble model (XGBoost) trained on data consisting of observations from individuals each with a trait $y_i$ and features $\mathbf{x }_i$, to rank features by importance using SHAP values. The ranked list of features makes it possible to construct new models that use only the most important features, and therefore have higher predictive power. Finally, having a fitted model that only consists of relevant features, we want to graphically present which relationships are important with respect to the phenotype, both marginal effects and interactions.

**Fig. 3:**
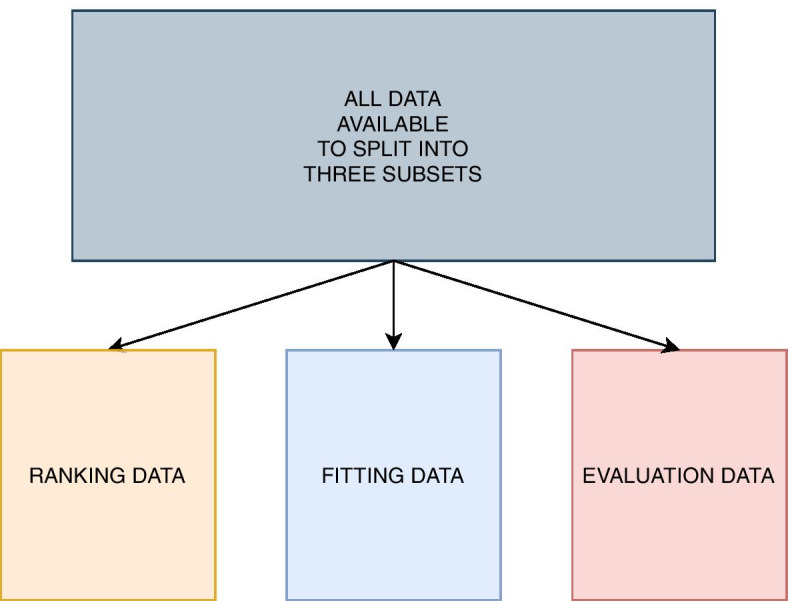
All data available is divided into three subsets: Ranking data, fitting data and evaluation data. The ranking data is used to rank features by importance in order to remove noise. The fitting data is used to fit models by using the ranking derived from the ranking data. The evaluation data is finally used to explain what is considered important with respect to the predictions from the models trained on the fitting data

In order to evaluate the ability to both rank features by importance, find the best predictive models, and explain the best models without causing optimism bias, we divide the individuals in three disjoint subsets, namely the *ranking data*, *fitting data* and *evaluation data* (Fig. 3).

Dividing the data into several subsets will reduce the power to detect relevant features as well as reducing the degree to which each subset is representative of the full data set. However, the procedures are intended to be used on data from large biobanks to reduce power loss and representativeness of the subsets. By using independent subsets of the data for each phase of our method, we avoid potential overfitting by reusing data, and will be able to give an accurate account to which extent tree ensemble models are able to capture relationships between features and the trait of interest that classical GWAS methods might have difficulties to achieve [40].

#### Phase 1: The ranking process

Identifying associations between SNPs and a phenotype is a typical example of a high-dimensional problem. Experience from several GWAS suggests that many low-effect SNPs are not detected. At the same time we still expect only a small proportion of the total genome to have any effect with respect to the trait of interest. Consequently, we face a challenge where many potential SNPs have a causal effect on the trait, but a much larger number of SNPs are not causal at all and therefore contribute as noise. To make it even more complicated, among the large number of SNPs in the human genome, there exist correlations between different SNPs throughout the whole genome in a given population called *linkage disequilibrium* [41]. In general, the closer the physical distance between a pair of SNPs is, the more correlated the SNPs tend to be. As not all SNPs are genotyped, and if we disregard imputed data, there will be gaps between the SNPs that are present. We expect that in many cases, SNPs with causal effect fall in such gaps. But here we are helped by the linkage disequilibrium and the correlation between nearby SNPs. For practical purpose this means that a subset of all SNPs available can provide information beyond only those SNPs selected, but also those nearby SNPs that are in linkage disequilibrium. This also applies for interactions.

The analysis is further complicated by confounders such as population stratification and cryptic relatedness between individuals which can lead to spurious associations in our models [42]. Cross-validation is a model validation technique in which several models of identical structure are trained on different portions of the training data, and each model is evaluated on independent validation data. With respect to feature importance, a procedure with the purpose of preventing spurious associations, is to evaluate the importance of each feature based on all models constructed during cross-validation.

**Fig. 4:**
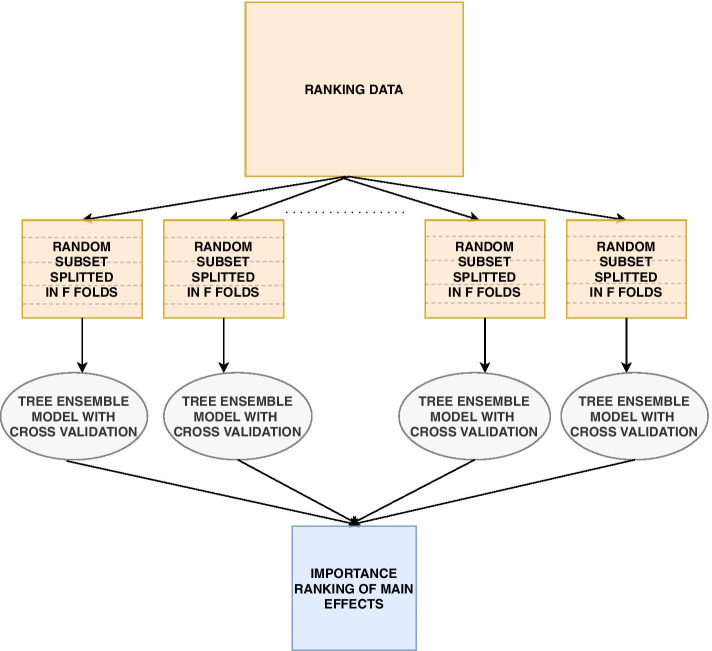
In the ranking process, multiple independent subsets are created and used in a cross-validation procedure with tree ensemble models. The trained models will be used to rank the importance of the features

From our knowledge about linkage disequilibrium, population stratification and cryptic relatedness, we therefore propose a method to implicitly investigate the whole genome efficiently and objectively through a series of independent cross-validations by using XGBoost, a tree ensemble model, as shown in Fig. 4. It is from these independent cross-validations we will provide a ranking of the importance of each feature.

**Fig. 5:**
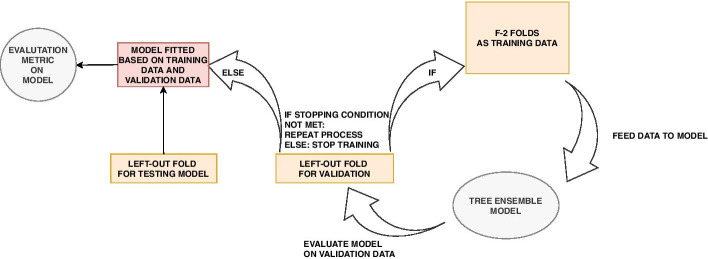
The cross-validation phase when training data consists of $F-2$ specific merged folds. Training of the model will proceed as long as the performance on the validation data improves within a certain number of iterations as given by the early_stopping_rounds hyperparameter

Consider a data set with *N* individuals and *R* directly genotyped SNPs. We create *A* randomly selected subsets, where each subset consist of *S* SNPs with low mutual correlation and $G \le N$ individuals randomly sampled with equal probability in order to keep an as agnostic narrowed search as possible. Furthermore, each sampled subset is divided into *F* folds where $F-1$ folds are used in an ordinary cross-validation to train $F-1$ XGBoost models, while the last fold never seen or used during cross-validation is used as test data. This will create $F-1$ models trained on different data, and their performance can be objectively evaluated on the test data. As shown in Fig. 5 for the $F-1$ folds used in cross-validation, in each iteration $F-2$ folds are used to train an XGBoost model, while the last fold is used as validation data. Training of the model will proceed as long as the performance on the validation data improves within a certain number of iterations as given by the early_stopping_rounds hyperparameter. Cross-validation reduces the harm of both overfitting and selection bias [43]. The degree of overfitting can be further investigated by looking at the model performance difference on the validation and test data.

With *A* subsets each creating $F-1$ models, the question is now how to rank all features investigated in all *A* subsets for all ${\mathcal {P}} = A(F-1)$ models. We define a new concept called the *relative feature contribution*, denoted $\kappa ^{p}_{i,j}$, for individual *i*, feature *j* and model *p* as:

6 $$\begin{aligned} \kappa ^{p}_{i,j} = \frac{|\phi ^{p}_{i,j}|}{|\phi ^{p}_{0}| + \sum _{m = 1}^M |\phi ^{p}_{i,m}|}, \end{aligned}$$

where $\phi ^{p}_{i,j}$ is the SHAP value for feature *j*. The measure $\kappa ^{p}_{i,j}$ can be interpreted as the proportion of the prediction for individual *i* attributed to feature *j* for model *p*. We now want to estimate the expected relative contribution of feature *j* using all the past independent cross-validations. The expected relative feature contribution (ERFC), ${\hat{E}}[\kappa _j]$, is defined as:

7 $$\begin{aligned} {\hat{E}}[\kappa _j] = \frac{1}{ \sum _{p=1}^{{\mathcal {P}}} G_p I(j \in \sigma _p) }\sum _{p=1}^{{\mathcal {P}}} \sum _{i = 1}^{G_p} I(j \in \sigma _p) \kappa ^{p}_{i,j}, \end{aligned}$$

where $\kappa ^{p}_{i,j}$ denotes the relative feature contribution of feature *j* for individual *i* in a set of $G_p$ individuals used to explain model *p*, and $I(j \in \sigma _p)$ is the indicator function which is equal to one if feature *j* is included in the subset data used to train model *p*, and zero elsewhere.

The individuals $G_p$ used to explain a particular model *p* created from a particular subset *a* are chosen to be the individuals from the test data of the subset. This means that the contribution of each feature in each model will be based on individuals never seen during training. The estimation of ${\hat{E}}[\kappa _j]$ for each feature *j* will finally create a ranking of the contribution of each feature.

#### Phase 2: The model fitting process

Given a ranked list of features based on their feature contribution with respect to the trait of interest, this allows us to disregard irrelevant features and thus increases the ability to detect important relationships.

**Fig. 6:**
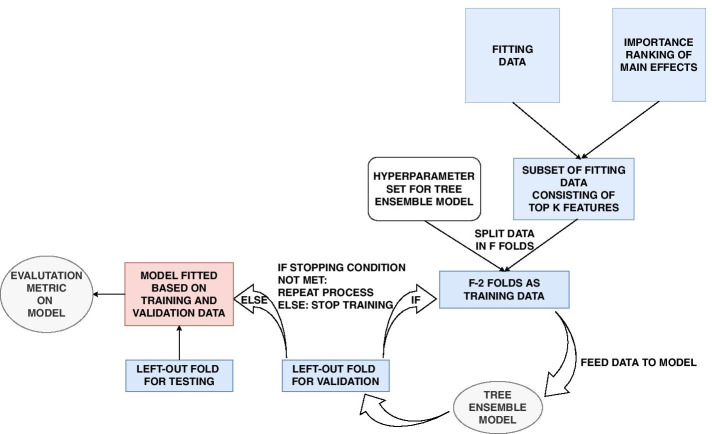
Given a table of ranked feature importances, XGBoost models based on the top *K* features are trained in a new cross-validation procedure based on an independent set of individuals, namely the fitting data. We search for the XGBoost models that on average performs the best for a given set of hyperparameters (including the value of *K*) based on test data

At this stage we are interested in finding the models with the best performance on some test data by utilizing the ranking of feature importance from the ranking process. For this purpose we use the fitting data never seen before in order to avoid any optimism bias [40]. The heterogeneity as well as possible relatedness among the individuals are taken into account by again using cross-validation. First we split the data in *F* folds, of which $F-1$ folds are used for cross-validation while the last fold is used as test data. This gives $F-1$ fitted models in total. The model fitting procedure is summarized in Fig. 6 which shows how one model (out of $F-1$) is fitted using only the top *K* features as well a set of hyperparameters. The aim is to find which set of $F-1$ models that on average performs best on the test data as a function of the value of *K* and hyperparameter values.

In order to explain the XGBoost models at a later stage we want to compute the SHAP values. We assume the features are mutually independent when computing the SHAP values. To take this into account, we combine the ranking with low values of the mutual squared Pearson’s correlation, denoted $r^2$, when selecting the *K* features to include. See Sect. 2 in Additional File 1 for more information. Even though we are not guaranteed an independent set of features using $r^2$, it significantly limits the number of dependent features and therefore reduces the negative effect of misleading computations of SHAP values.

#### Phase 3: Model explainability

After finding the best predictive models from the model fitting process, we can investigate which features and interactions contribute to the models through the SHAP values. Along the same lines as for the marginal feature importance used for ranking, the *relative contribution* for each interaction between feature *j* and *k* for a particular individual *i* and model *p* can be computed as:

8 $$\begin{aligned} \mu ^{p}_{i,j,k} = \frac{2|\Phi ^{p}_{i,j,k}|}{|\phi ^{p}_{0}| + \sum _{m=1}^M |\phi ^{p}_{i,m}| } \end{aligned}$$

We can estimate the expected relative interaction contribution, ${\hat{E}}[\mu ^{p}_{j,k}|G_e,p]$, given data consisting of $G_e$ individuals and a model *p*:

9 $$\begin{aligned} {\hat{E}}[\mu ^{p}_{j,k}|p,i=1,...,G_e] = \frac{1}{G_e} \sum _{i=1}^{G_e} \mu ^{p}_{i,j,k}. \end{aligned}$$

The $G_e$ individuals are part of the evaluation data as shown in Fig. 2. As we have $F-1$ models from the model fitting process, we average the result from all $F-1$ models:

10 $$\begin{aligned} {\hat{E}}[\mu ^{p}_{j,k}] = \frac{1}{F-1} \sum _{p=1}^{F-1} {\hat{E}}[\mu ^{p}_{j,k}|p,i=1,...,G_e]. \end{aligned}$$

We define this new concept as the expected relative interaction contribution (ERIC). This will provide a ranked list of interactions. A ranked list of marginal effects can be constructed as explained in the ranking process, but this time based on the $F-1$ models constructed after the model fitting process.

The contribution of the top ranked marginal effects and interactions to the prediction for each individual can be visualized with sina plots and partial dependence plots as illustrated in Figs. 10 and 11 [17]. For one particular trained tree ensemble model, the sina plot in Fig. 10 shows the SHAP value for each individual indicated as a point with color depending on the value of the feature. The larger the absolute SHAP value, the more the feature contributes to the model prediction for a specific individual. Partial dependence plots, exemplified in Fig. 11, are used to visualize how the contribution, in other words the SHAP value, for a particular feature depends on another feature for different combinations of feature values. Here as well, each individual is marked as a point with the value of a given feature given on the x-axis and the corresponding SHAP value for this feature with respect to the prediction on the y-axis. The color of the point, however, represents the value of some other feature. In this way, interactions can be visualized and interpreted.

## Results: application using UK Biobank data

As an example, we apply and evaluate the method described on data from the UK Biobank Resource [44]. Among the available phenotypes, obesity was chosen because it has been subjected to a number of high quality and well-powered GWAS that have identified more than 100 loci, many that have been consistently replicated across studies (e.g. FTO, BDNF, MC4R, TMEM18, SEC16B) [18–20] . Thus, we have a good set of true-positive loci with which to compare our results. We only analyzed White European individuals to limit the effect of population stratification. We define an individual to be part of either the control group ($y_i =0$) or case group ($y_i=1$) by:

11 $$\begin{aligned} y_i= {\left\{ \begin{array}{ll} 1,&{} \text {if } 30 \le {{\,\mathrm{BMI}\,}}\le 70\\ 0,&{} \text {if } 18.5 \le {{\,\mathrm{BMI}\,}}\le 25 \end{array}\right. } \end{aligned}$$

As should be evident above, we exclude overweight individuals with $25< {{\,\mathrm{BMI}\,}}< 30$ from the analysis and only compare normal-weight individuals ($18.5\le {{\,\mathrm{BMI}\,}}\le 25$) with obese individuals (${{\,\mathrm{BMI}\,}}\ge 30$). This reduces the number of subjects available for analysis, but allows us to define more distinct case and control groups. For power analyses of extreme phenotype data we refer the reader to [45]. The BMI data is provided from measurements at the initial assessment visit (2006–2010) at which participants were recruited and consent given. Phenotype-independent quality control of the genetic data for White European subjects consisting of the genotyped SNPs is completed using PLINK1.9 [46], and the details are given in Additional File 1. We only consider directly genotyped SNPs. In addition, we limit our analysis to SNPs with minor allele frequency (MAF) greater than 0.01. By only considering the two groups defined in Equation (11), this results in a total of 529 024 SNPs and 207 015 individuals to investigate, of which 43% of these individuals are in the group defined as obese. We apply the R package xgboost to both train xgboost models and to estimate SHAP values [47].

### Environmental features

We include environmental features that are previously reported to be of importance with respect to obesity, namely sex, age, physical activity, intake of saturated fat, sleep duration, stress and alcohol consumption [48–52]. These environmental features are a representative set for the demonstration of the methodology and were not intended to be an exhaustive set of environmental features available in the UK Biobank for obesity. Information about the environmental features, including their definitions, are included in Additional File 1.

### Ranking, fitting and evaluation data

We let the ranking data consist of 80,000 randomly chosen individuals, which will be used to rank the features by importance. The fitting data also consists of 80,000 individuals. This subset is used to find the best predictive models in the model fitting process. The evaluation data consists of 47 015 individuals, and is used to explain what the models found in the model fitting process consider the most important features and in which way they contribute. In all subsets, we retain the proportion of obese individuals.

### Phase 1: The ranking process

By using the ranking data, at this stage we create $A=50$ subsets where each subset consists of $G = 70{,}000$ individuals and $S=110{,}000$ randomly chosen SNPs corresponding to 21% of the total number of SNPs available. The choice of total number of subsets to create is motivated from Eq. (2) in Additional File 1 with the criteria that any pair of SNPs appears in the same subset at least once with 90% certainty. The larger the number of individuals in each subset, the higher statistical power, but at the same time, the memory capacity limits the number of individuals in each subset at the cost of lost power. As the ranking process is time-consuming, we do not attempt any sophisticated hyperparameter optimization, but instead choose four hyperparameters sets that we regard as reasonable, given in Table 1. In addition, in all further analysis, the regularization parameter $\lambda$ is set to 1, the default value in most XGBoost softwares [47]. The parameter early_stopping_rounds is set to 20.

As discussed in Blagus and Lusa [31], the learning rate $\eta$ is set to be small for high-dimensional data such as 0.1, while as discussed in Chen and Guestrin [16], colsample_bytree is set to be large as there is only a small proportion of all features that are relevant. The hyperparameter subsample is also set to be large in order to increase the power to detect features of importance. The parameter colsample_bylevel has not been extensively discussed in the literature, but the parameter will oppose the greedy construction of the trees which may be beneficial in the long run. The maximum depth of the trees are set to no more than three, the reason being both computational considerations as well as the fact that the marginal expectations used to compute the SHAP values in (3) will be more inaccurate the deeper the trees are (see Additional File 1).

**Table 1:** The four hyperparameter sets for XGBoost considered in the analysis during the ranking process

Set	${\eta }$	colsample_bytree	subsample	colsample_bylevel	max_depth
1	0.01	0.9	0.9	0.9	2
2	0.05	0.8	0.8	0.8	2
3	0.05	0.8	0.8	0.8	3
4	0.1	0.8	0.8	0.8	2

Using Eq. (7) to estimate the expected relative contribution for each feature, we give the ranking for the top 20 features in Table 2 for hyperparameter set 2 in Table 1.

**Table 2:** The resulting ranking based on the expected relative feature contribution (ERFC) from the ranking process for hyperparameter set 2 in Table 1

Feature	ERFC
Sex	0.12
Alcohol intake frequency	0.12
Physical activity	0.11
Saturated fat intake	0.058
Stressful events	0.056
Sleep duration	0.049
Age at initial assessment	0.047
rs17817449 (FTO, Chr. 16)	0.025
rs1421085 (FTO, Chr. 16)	0.025
rs1121980 (FTO, Chr. 16)	0.024
rs7202116 (FTO)	0.023
rs9941349 (FTO)	0.023
rs9940128 (FTO)	0.023
rs9922619 (FTO)	0.023
rs13393304 (FAM150B - TMEM18, Chr. 2)	0.022
rs12149832 (FTO)	0.021
rs9939609 (FTO)	0.021
rs9930506 (FTO)	0.021
rs11642841 (FTO)	0.020
rs2947411 (Chr. 2)	0.019

The environmental features are, as expected, considered more important than the SNPs, while the most important SNPs are at or nearby the FTO gene in agreement with previous studies

Not surprisingly, the environmental features are considered most important. The next features are predominantly those connected to the FTO gene at chromosome 16 as expected from previous studies. A SNP close to the TMEM18 gene (rs13393304) is also found in the top 20 list. The next SNPs on the list are predominantly from chromosome 2, one SNP from chromosome 1 at the SEC16B gene (rs10913469) and further down SNPs from chromosome 18, yet no SNPs connected to the MC4R gene for instance. By further investigation, this is due to the fact that the SNPs randomly selected from the 50 subsets did not include any SNPs close to the MC4R gene which illuminates the issue when not creating enough subsets. Apart from this, one can see that the ranking process is able to detect small effects, and importance of each feature can be evaluated by computing SHAP values.

We compare with the corresponding ranked list derived using BOLT-LMM, a Bayesian mixed model that evaluates the marginal effect of each SNP, and computes *p*-values based on the BOLT-LMM infinitesimal mixed-model statistic [1]. The *p*-values are shown to be well-calibrated for significance levels as low as $5 \cdot 10^{-8}$ when the MAF of each SNP is larger than 1%, and that the case fraction is larger than 30% for a sample of 50,000 individuals [53]. All these criteria are satisfied in our ranking data set (with case fraction 42%, MAF greater than 1% and 80,000 individuals). Table 3 shows the top ranked 13 SNPs (top environmental features are not listed) where features with the smallest *p*-values are regarded to be of most importance.

**Table 3:** The result after running BOLT-LMM on the ranking data showing the top SNPs with smallest *p*-value from the BOLT-LMM infinitesimal mixed-model statistic

Feature	BOLT-LMM *p*-value
rs1421085 (FTO)	3.7E–57
rs9940128 (FTO)	1.8E–54
rs1121980 (FTO)	2.4E–54
rs3751812 (FTO)	7.0E–54
rs17817449 (FTO)	8.5E–54
rs9939609 (FTO)	1.3E–53
rs8050136 (FTO)	2.2E–53
rs7202116 (FTO)	5.7E–53
rs9941349 (FTO)	5.0E–52
rs12149832 (FTO)	3.0E–50
rs9922619 (FTO)	1.0E–48
rs9930506 (FTO)	1.1E–48
rs11642841 (FTO)	1.3E–40

All top SNPs are connected to the FTO gene

In this case, all SNPs are related to the FTO gene, and most of the SNPs except two are also present in Table 2. These two SNPs were not sampled in any subset from the ranking process. The ordering in Table 2 and 3 between SNPs related to the FTO gene are slightly different. However, at this stage it is not strictly necessary to find the true order of the feature impacts, but an approximate order that allows us to discard features with insignificant impact in the further analysis.

### Evaluation of the trained models used in the ranking process

**Fig. 7:**
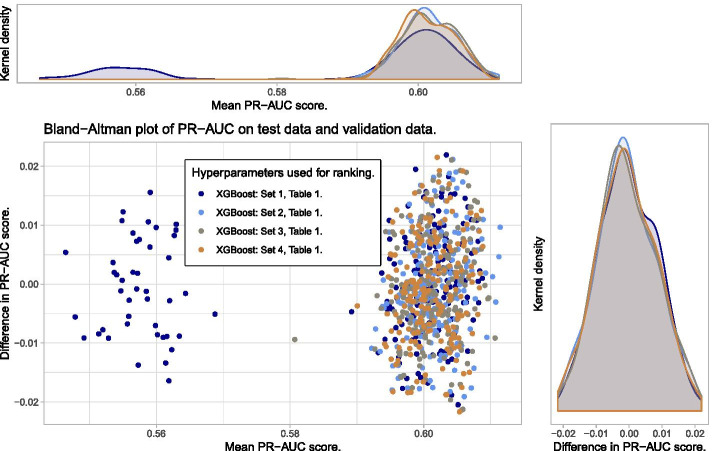
Bland–Altman plot for the trained models used for ranking. No clear signs of overfitting, but one set of hyperparameters shows one cluster of poorer predictions than the others

To explore the degree of overfitting of the models trained during the ranking process, the PR-AUC score of each model computed on its corresponding validation data and test data (see Fig. 5) are explored in a Bland–Altman (mean—difference) plot. This shows the average PR-AUC score for each model on the x-axis, and the difference between the two scores on the y-axis. The results for all chosen sets of hyperparameters given in Table 1 can be seen in Fig. 7.

**Fig. 8:**
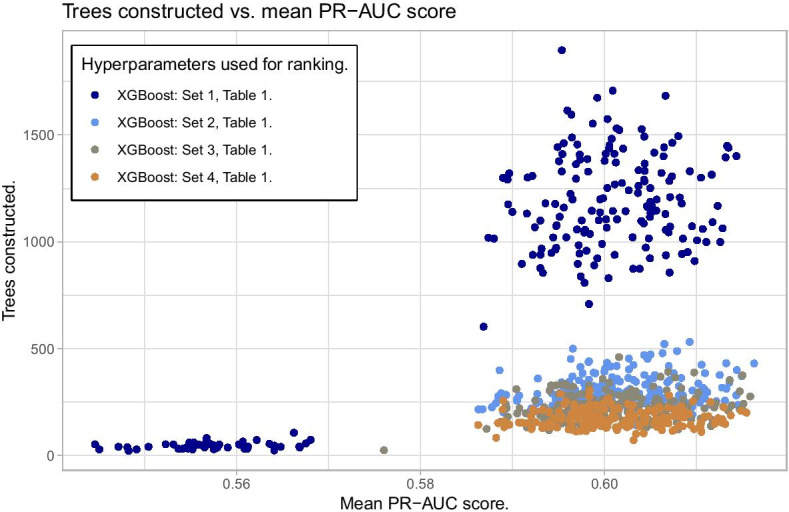
The reason some models with hyperparameter set 1 in the ranking process underperforms is early stopping of the training. Notice also that a larger number of trees need to be constructed to get the same performance as for models with other hyperparameter sets

Figure 7 shows no clear pattern of overfitting as can be seen from the agreement between the density plots of the difference in PR-AUC scores. However, hyperparameter set 1 from Table 1 shows a cluster of bad predictions with PR-AUC around 0.56. The reason for this can be seen in Fig. 8 where bad predictions using hyperparameter set 1 is due to early stopping in the training. When there is no early stopping in the training, we also see that due to the small learning rate given in set 1, more trees are constructed than for the other hyperparameter sets, but yet the performance score is not superior. This emphasizes the importance of hyperparameters.

### Phase 2: model fit from the ranking process and from BOLT-LMM ranking

In the model fitting process, we use the fitting data to train new XGBoost models with cross-validation by including the *K* most important SNPs for $K=0$ (only including environmental features), $K=100$, 500, 1000, 3000, 5000, 10,000 and finally $K=15{,}000$. The ranking of the features is the output of the ranking process. In addition, to assess the quality of our method, we also train models based on the ranked table produced by BOLT-LMM.

**Fig. 9:**
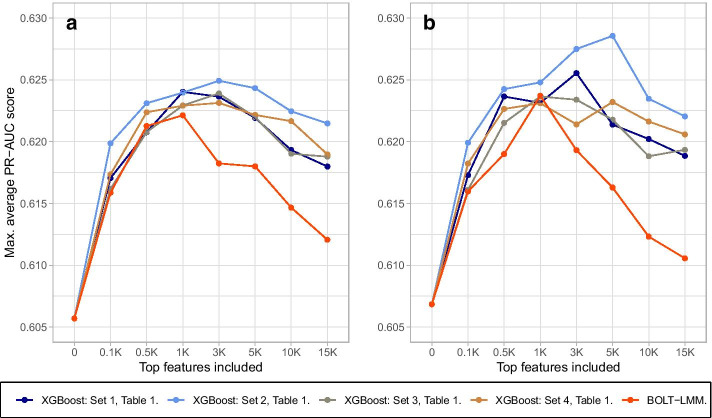
The model fitting process based on top *K* features from both the ranking process (for different sets of XGBoost-hyperparameters indicated by the different colours and the legend) and from BOLT-LMM, for different values of *K*. In **a** hyperparameter sets 1–8 (all with $\hbox {max}\_{\mathrm{depth}}=2$) from Table 4 in the model fitting process are used. In **b** hyperparameter sets 9–16 ($\hbox {max}\_{\mathrm{depth}}=3$) are used. Both figures show that the use of the ranking process gives in general better model performance than for the BOLT-LMM ranking. There is also some gain in performance by increasing the hyperparameter max_depth from two to three

Before training, the set of the *K* chosen SNPs is reduced such that the SNPs have mutually squared Pearson’s correlation $r^2 < 0.2$ (see Additional File 1 for practical details about implementation). Due to computational limitations, we will only consider hyperparameter tuning from the XGBoost models through the sets given in Table 4, and optimize based on these sets. For each *K* and for the ranking based on our method and the ranking based on the BOLT-LMM model, the maximum average PR-AUC-score for the XGBoost models constructed in the cross-validation is found among the possible hyperparameter sets. For each *K*, we compare how the predictive model perform on the held-out test data from the fitting data. The results are shown in Fig. 9. When we vary *K* from small to large values, we expect that the model performance increases the most at the beginning as the most influential features are included, while as more features with low importance are added, the performance increases steadily until it flattens. At the end, the performance may even decrease as noise are added to the model in the form of SNPs without any predictive power.

**Table 4:** The hyperparameter sets considered during the model fitting process

Set	$\varvec{\eta }$	colsample_bytree	Subsample	colsample_bylevel	max_depth
1	0.1	0.3	0.3	0.3	2
2	0.1	0.5	0.5	0.5	2
3	0.1	0.5	0.5	1	2
4	0.1	0.8	0.8	0.8	2
5	0.1	1	1	1	2
6	0.05	0.5	0.5	0.5	2
7	0.05	0.8	0.8	0.8	2
8	0.2	0.5	0.5	0.5	2
9	0.1	0.3	0.3	0.3	3
10	0.1	0.5	0.5	0.5	3
11	0.1	0.5	0.5	1	3
12	0.1	0.8	0.8	0.8	3
13	0.1	1	1	1	3
14	0.05	0.5	0.5	0.5	3
15	0.05	0.8	0.8	0.8	3
16	0.2	0.5	0.5	0.5	3

The turning point for the BOLT-LMM ranking is $K = 1000$ while for the models based on the ranking process the turning point is consistently for a larger *K* value. The maximum average PR-AUC-score for the XGBoost models created in cross-validation is in general larger when using the ranking based on our method than the ranking based on BOLT-LMM. From Fig. 9, the average performance score is in general better when allowing the regression trees to be of maximum depth three instead of two. Additionally, inclusion of the SNPs provide only a small contribution to the increase in the average prediction performance, where the best models increase the average PR-AUC score from 0.606 when only environmental features are included to 0.629 when the top 5000 SNPs are included (blue line, Fig. 9b). This corresponds to an increase in average classification accuracy from 0.64 to 0.66.

### Phase 3: Model explainability

**Fig. 10:**
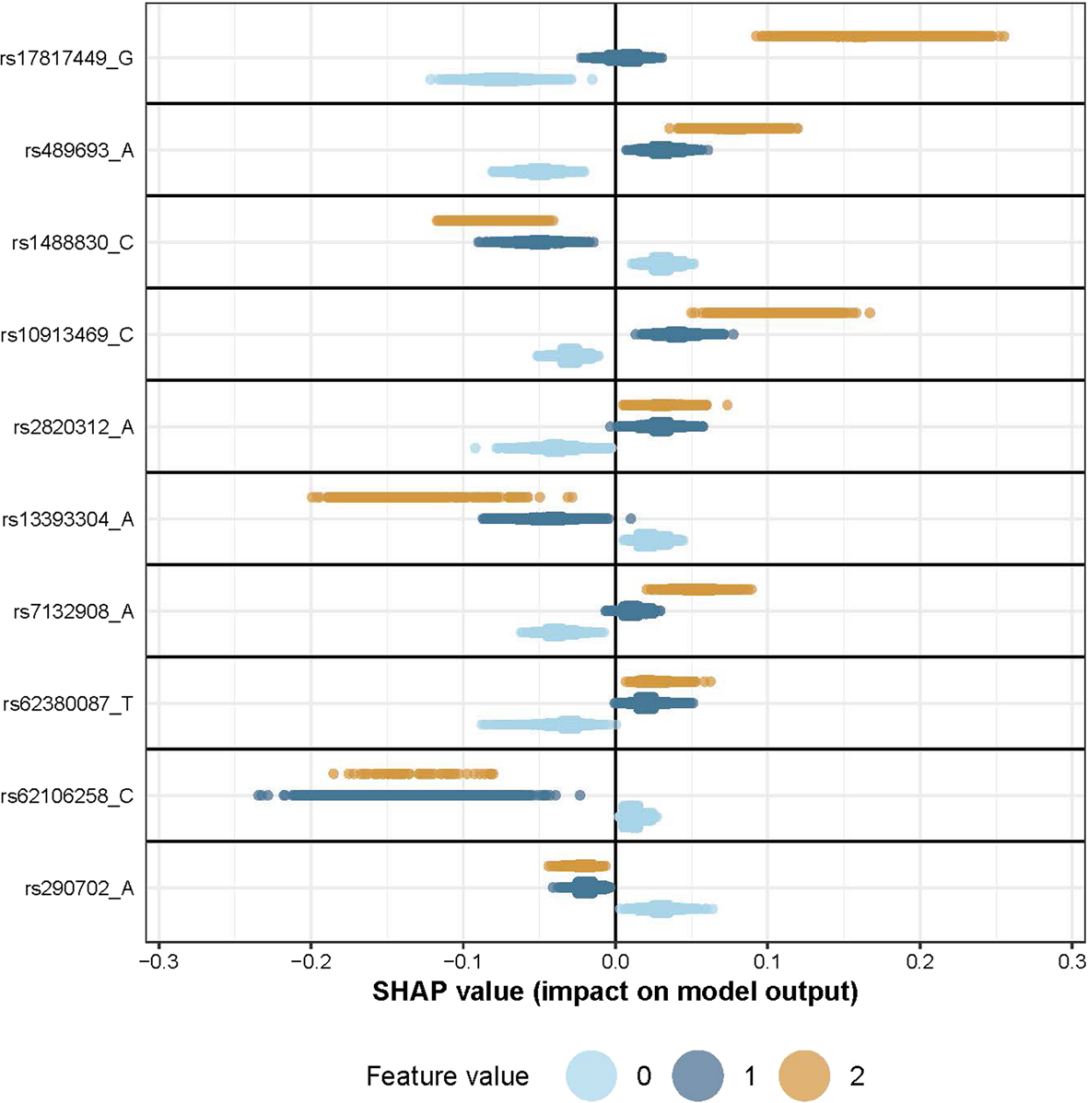
A sina plot visualise the importance of each feature from a fitted model. Here we show the sina plot of the marginal effects for one of the four models constructed during the model fitting process when applied to the evaluation data from UK Biobank

In the model explainability phase we use the evaluation data consisting of 47,015 individuals, that has not been used in Phase 1 and 2. For convenience, we consider the models constructed during cross-validation that performed best on average on the test data during the model fitting process. These are the four models from fourfold cross-validation trained on the top 5000 ranked features with hyperparameter set 2 visualised as the blue line in Fig. 9b. We now explore what these four models consider important with respect to their predictions on the evaluation data. This is done by computing the expected relative contribution for both individual features as well as interactions. Marginal and interaction effects can be visualized with sina plots and partial dependence plots respectively. For the case of marginal effects, Fig. 10 shows the sina plot for one of the four models trained on the SNPs with the largest expected relative contributions. Here, we visualize both dominant and additive main effects found by our nonparametric method.

We use Eq. (8) together with Eq. (10) to compute the average relative interaction contribution (ERIC) for each pair of features based on the evaluation data, and list the top 10 interaction candidates in Table 5.

**Table 5:** The top 10 interactions based on the expected relative interaction contribution (ERIC) estimated on the evaluation data (Phase 3), with the aim of explaining the best predictive models from Phase 2

Feature 1	Feature 2	ERIC
rs171329	rs180743	0.001
Sex	rs17817449	0.001
Saturated fat intake	rs17817449	0.00094
rs757318	rs12123815	0.0008
rs4697952	rs1488830	0.00074
rs60822591	rs17854357	0.00066
rs4711329	rs11676272	0.00066
rs1518278	rs1488830	0.0006
Sex	rs12123815	0.00056
rs7132908	rs9949796	0.00054

**Fig. 11:**
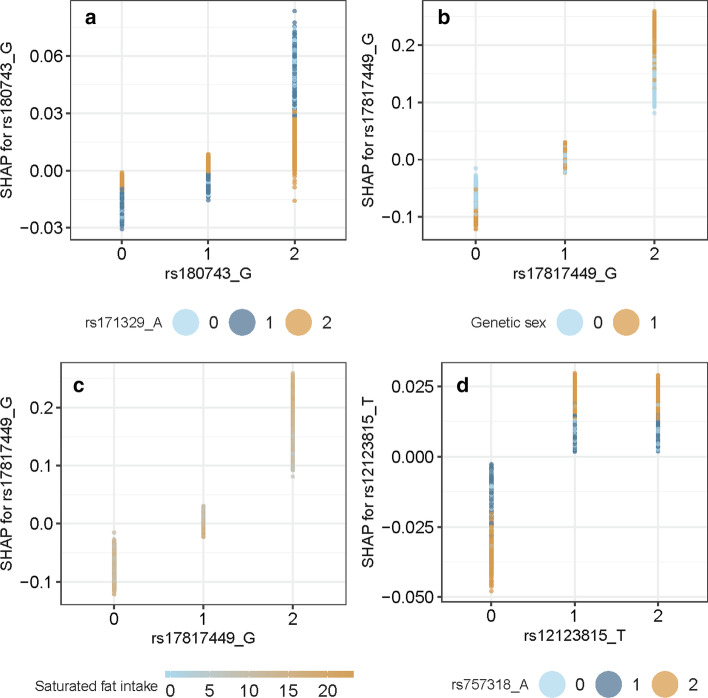
Partial dependence plots for the pairs **a** rs180743 and rs171329, **b** rs17817449 and genetic sex, **c** rs17817449 and saturated fat intake, and **d** rs12123815 and rs12123815. In all panels we see how the SHAP values (vertical axis) depends on the feature value of the SNP (horizontal axis) and on the value of the second feature (color)

First of all, we see that the contributions from the interactions are quite small with expected relative interaction contribution (ERIC) of no more than 0.001. To further investigate the behaviour of these interaction candidates, in Fig. 11 we show partial dependence plots [17, 26] for the top four interactions from Table 5 when regarding one specific chosen model, out of the four, for each interaction.

We see in Fig. 11 examples where the SHAP value of the feature for each individual represented along the x-axis not only depends on its own feature value, but the value of some other feature as well. For instance, in Fig. 11a, we see that the increased risk of being obese when the genotype value is equal to two for rs180743, is reduced if the genotype value of rs171329 is equal to two as well. We also see in Fig. 11b that being a male (orange points) gives higher risk of being obese when the genotype value of rs17817449 is two, compared to when the genotype value is zero or one. A positive SHAP value implies a positive contribution to the log-odds prediction, and therefore a contribution making it more likely to be a case (obese).

### Interaction models in logistic regression

We compare the interaction rankings from Phase 3 with logistic regression fits on the full UK Biobank data set and the evaluation data alone. We consider a parametric model, assuming additive effects, for both SNP-SNP and SNP-environment interaction effects for logistic regression, and construct a hypothesis test to infer the presence of interactions. For the test of SNP-SNP interactions between two SNPs *a* and *b*, the null model will be:

12 $$\begin{aligned} {{\,\mathrm{logit}\,}}_{H_0,add}(P(Y_i=1|g_{i,a},g_{i,b},\mathbf{x }_{i,c})) = \mathbf{x }_{i,c}^{T}\gamma + \alpha g_{i,a} + \beta g_{i,b}, \end{aligned}$$

where $\mathbf{x }_{i,c}^{T}$ is a vector of features such as intercept, age, environmental features and principal components, while $\gamma$ is the vector of corresponding parameters for each feature. The parameters $\alpha$ and $\beta$ are the marginal effects from SNP *a* and *b* resepectively. The corresponding alternative model incorporating an additive interaction effect will be:

13 $$\begin{aligned} {{\,\mathrm{logit}\,}}_{H_1,add}(P(Y_i=1|g_{i,a},g_{i,b},\mathbf{x }_{i,c})) = \mathbf{x }_{i,c}^{T}\gamma + \alpha g_{i,a} + \beta g_{i,b} + \nu g_{i,a} g_{i,b}. \end{aligned}$$

For a SNP-environment interaction we will use the following alternative model:

14 $$\begin{aligned} {{\,\mathrm{logit}\,}}_{H_1}(P(Y_i=1 |g_i,x_{i,e},\mathbf{x }_{i,c})) = \mathbf{x }_{i,c}^{T}\gamma + \alpha g_{i,a} + \beta _e x_{i,e} + \phi g_{i,a} x_{i,e}, \end{aligned}$$

where $\beta _e$ and $\phi$ are marginal environmental effect and interactions parameters respectively.

For the testing of the interactions we apply the likelihood ratio test (LRT) to test the null hypothesis that $\nu = 0$ for SNP-SNP interactions or $\phi = 0$ for SNP–environment interactions [26, 54]. The LRT assumes independence between the samples, and so we need to make sure the individuals included in the test are not related to any significant degree.

#### Comparison of Phase 3 results with logistic regression tests

Let the vector $\mathbf{x }_{i,c}$ given in (13) consist of the intercept in addition to the features sex, age and the top four principal components for each individual. The principal components are used to correct for population stratification [55]. The ranking of the pairwise interactions is based on the evaluation data consisting of 47,015 individuals. We fit a logistic regression model based on all unrelated individuals in the evaluation data (39,286 individuals), as well as a logistic regression based on all unrelated individuals used in this paper (173,468 individuals). Unrelatedness is ensured by using data field 22020 in the UK Biobank Data Showcase [44]. The principal components were calculated using EIGENSOFT (version 6.1.4) SmartPCA [56, 57]. We compute the principal components on the unrelated individuals in the evaluation data and all unrelated individuals separately. PCA plots for both the evaluation data and the full data set can be seen in the Additional File 1. A few individuals have missing values for each test and are removed.

The top four interactions from the SHAP values visualized in Fig. 11 are evaluated by applying likelihood ratio tests for each interaction. The results are given in Table 6.

**Table 6:** Results from likelihood ratio tests applied on the top four ranked interactions found from the model explainability process based on the evaluation data

Data set	Interaction	*p*-value LRT
Evaluation data	rs171329 and rs180743	0.85
All individuals	rs171329 and rs180743	0.024
Evaluation data	rs17817449 and genetic sex	0.77
All individuals	rs17817449 and genetic sex	4.09e-05
Evaluation data	rs17817449 and saturated fat intake	0.44
All individuals	rs17817449 and saturated fat intake	0.0017
Evaluation data	rs757318 and rs12123815	0.25
All individuals	rs757318 and rs12123815	0.71

It is clear that the sample size is the dominating factor for the computed *p*-values. All *p*-values based on the evaluation data, the same data that is used to rank the interactions, are non-significant. As expected, the *p*-values are in general smaller when considering all individuals, yet none of them would be declared significant in the case of any reasonable genome-wide multiple testing procedure [58]. The smallest *p*-value is achieved for the interaction between the SNP rs17817449 and genetic sex when including all individuals. In the Additional File 1, we apply likelihood ratio tests based on logistic models with less stricter assumptions, but with the need for more parameters. However, this does not provide smaller *p*-values to any significant degree. The reason may be that these tests are less powerful due to a higher number of degrees of freedom [54].

### Stratified analysis

**Fig. 12:**
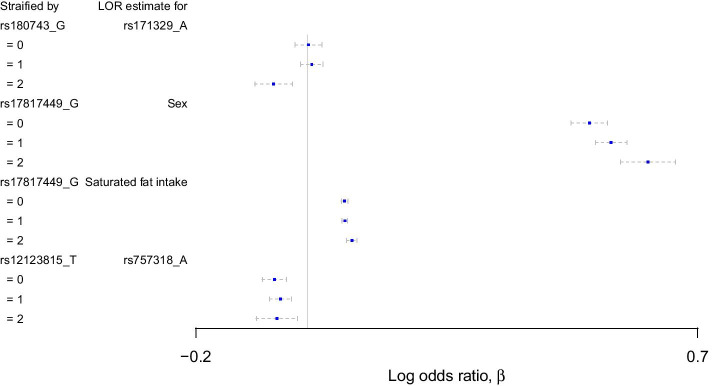
Stratified analysis of the top four interactions based on all unrelated individuals to illustrate how the log odds ratio, with 95% confidence intervals, of one feature changes depending on the value of another feature

Instead of incorporating prespecified interactions in the logistic regression model, one can instead stratify in groups according to the value of a feature *a*, and investigate the effect of a feature *b* for each group. For instance, one can fit for each group a logistic regression model with respect to feature *b* such as in (12). For a true interaction, the log odds ratio of feature *b* will differ between some or all groups. Fig. 12 shows a stratified analysis for the top four interactions in Table 5, with 95% confidence intervals assuming normality of the estimated log odds ratios, adjusting for the same environmental features. The first example where the log odds ratio of rs171329 is compared within stratified groups of rs180743 do not change additively, the opposite of what is assumed in (13). However, the second example concerning rs17817449 and sex do show additive changes in the log odds ratios. The third interaction also shows small, yet indicative, differences in the log odds ratios. In the last example with rs12123815 and rs757318 the uncertainties in the log odds ratios are too large to give any conclusion.

## Discussion

We have proposed how tree ensemble models, such as XGBoost, can be combined with SHAP values to explain the importance of individual SNPs as well as gene–gene and gene–environment interactions. The method has been illustrated on an example from the UK Biobank. We have shown that through several independent cross-validations on XGBoost models using subsets of SNPs spread along the genome, one is able to find a reasonable ranking of individual SNPs similar to what is found in previous GWAS of obesity [18]. In fact, Fig. 9 suggests that the ranking process has the potential to outperform BOLT-LMM.

### Ranking of interactions through SHAP values

The SHAP values may also identify interactions, but further investigation is needed. Comparing the top ranked interactions with logistic regression including interaction parameters, we see that none of the corresponding statistical tests provide convincing *p*-values. Assuming the ranking of interactions via SHAP values is reliable, we see from Table 5 that the interaction effects are small. Any genome-wide multiple testing procedure would struggle to find such small interaction effects. In addition, misspecification of the effects in the logistic regression models may reduce statistical power. Figure 12 shows that only the potential interaction between rs17817449 and sex seems to be additive. Tree ensemble models do not have any presumptions of what kind of effects are present, but instead they learn the effects iteratively. These effects can be investigated efficiently through SHAP values. However, the SHAP values are estimated, and uncertainties in these estimates must be accounted for. The interaction between rs12123815 and rs757318 in Fig. 12 is an example that may very well be a false positive. There is therefore a need to develop tests that can infer the trustworthiness of the SHAP values in a similar fashion as through *p* values. The development of such tests will be important future research within SHAP values.

One natural way to account for some of the uncertainties in the SHAP values is through cross-validation. In addition, larger absolute SHAP values may not only be as a consequence of larger feature importance, but also as a consequence of larger uncertainties in the SHAP values. The denominator in ERFC and ERIC, given in (7) and (10), equal to the sum of the absolute SHAP values for each individual will tend to be larger, the larger the variance of the SHAP value estimates are. Consequently, the importance measures ERFC and ERIC are reduced for increasing uncertainties in the SHAP values.

### Data split

In this paper, data is split in three subsets used for ranking, model fitting and model explanation respectively. This procedure requires a large amount of data, but the purpose was to evaluate the credibility and potential of using tree ensemble models together with SHAP values. For smaller data samples, an alternative procedure is to rank interactions directly during the ranking process by computing the expected relative interactions contributions (ERIC). However, the ranking process consists of many models with low predictive power, which makes it more difficult to explore the true relationships compared to the models constructed in the model fitting process.

### Limitations and improvements

The choice of number of SNPs *S*, individuals *G*, folds *F* and $r^2$-threshold in each cross-validation in the ranking process are all important with respect to performance, and should be considered as hyperparameters. The number of SNPs *S* must be large enough to represent important regions in the genome, but not so large that it introduces noise to the model. The number of individuals in each cross-validation, *G*, should be as large as possible as it increases the power to detect small as well as nonlinear effects. However, that may lead to computational challenges. The number of folds in the cross-validations, *F*, should neither be too small nor too large as we want to train the model on as many different subsets of the population as possible in order to find the most general effects, but at the same time the validation data set must be large enough to be sufficiently representative.

The mutual independence assumption when computing the SHAP values is a significant restriction, and a mutual $r^2$ below any threshold between features will by no means ascertain mutual independence as $r^2$ measures linear dependency. Correlation measures that can also account for nonlinear dependencies in a high-dimensional setting could provide more trustworthy results.

### Hyperparameter optimization

We have seen that the hyperparameters for XGBoost are important. Unfortunately, the computation time for each set of hyperparameters is protracted, and consequently systematic hyperparameter optimization is not feasible. However, from the choice of hyperparameter sets in this paper, the hyperparameters colsample_bytree, subsample and colsample_bylevel should be high (0.8–0.9), while the learning rate $\eta$ should be low (0.05–0.1), but not too low. Another important hyperparameter, the regularization parameter, $\lambda$, should be investigated more extensively.

### Predictive performance and obesity

Even with strong predictors such as physical activity, intake of saturated fat, alcohol use, stressful events, sleep duration, age and sex in addition to genome-wide genetic data, we are not capable of constructing a model with more than 66% classification accuracy, and the genetic data only provide a small portion of the predictive performance. The usefulness lies in the fact that tree ensemble models can be used to identify nonparametric gene–gene and gene–environment interaction candidates while accounting for a large amount of features simultaneously. If the prediction performance of the model is considered satisfactory, this can be an important diagnostic tool in the future.

## Conclusion

Our proposed tree ensemble- and SHAP-based method gives us the possibility of exploring both gene–gene and gene–environment interactions without any presumptions of what kind of effects may be present as well as adjusting for environmental features. Our proposed method can be applied to high-dimensional genetic data in large-scale biobanks. There is however a need to develop methods for assessing the uncertainties of the SHAP values to conclude whether the interaction candidates are reliable.

## Supplementary Information

**Additional file 1.** Supplementary materials providing technical details about quality control of genetic data, theory, implementation as well as figures.

## Data Availability

The research has been conducted using the UK Biobank Resource under Application Number 32285. The application for access to the UK Biobank Resource was approved on October 10, 2018. Source code supporting this paper can be found online at https://github.com/palVJ/GWASwithTreeSHAP/.

## Abbreviations

BMI: Body mass index
ERFC: Expected relative feature contribution
ERIC: Expected relative interaction contribution
FN: False negative
FP: False positive
FWER: Family-wise error rate
GWAS: Genome-wide association study
LRT: Likelihood ratio test
MAF: Minor allele frequency
PCA: Principal component analysis
PR-AUC: Precision-recall area-under-curve
SHAP: Shapley additive explanation
SNP: Single nucleotide polymorphism
TP: True positive
