# Supplementary material for: A new method for exploring gene–gene and gene–environment interactions in GWAS with tree ensemble methods and SHAP values
Source: BMC Bioinformatics. 2021 May 4;22:230. doi: 10.1186/s12859-021-04041-7 (PMC8097909; doi:10.1186/s12859-021-04041-7)
Supplement: Supplementary file 1 — Additional file 1. Supplementary materials providing technical details about quality control of genetic data, theory, implementation as well as figures. [file 12859_2021_4041_MOESM1_ESM.pdf]

A new method for exploring gene-gene and gene-environment interactions in GWAS with tree ensemble methods and SHAP values
Supplementary File

Pål Vegard Johnsen[1,2], Signe Riemer-Sørensen[1], Andrew Thomas DeWan[3], Megan E. Cahill[3], and Mette Langaas[2]

[1]SINTEF Digital, Oslo, Norway
[2]Department of Mathematical Sciences, Norwegian University of Science and Technology, Trondheim, Norway
[3]Department of Chronic Disease Epidemiology and Center for Perinatal, Pediatric and Environmental Epidemiology, Yale School of Public Health

1 Quality assessment of UK Biobank Genetic Data

Analyses were limited to autosomal variants covered by both genotype arrays used over the course of the study and that passed the batch-level quality control. SNPs were included if the call rate was above 99%, the Hardy-Weinberg equilibrium p-value was less than $5 \cdot 10^{-8}$, and the minor allele frequency was larger than 1%. 529,024 SNPs passed these filters.

Individuals were removed if the genetic and reported sex did not match and if the sex chromosomes were not XX or XY. Outliers in heterozygosity and missing rates were removed. The analyses were limited to those identified as Caucasian through the UK Biobank's PCA analysis (field 22006). All individuals had an individual call rate larger than 99%. 366,752 individuals passed these filters.

2 Details of environmental features from UK Biobank

A sample set of personal and environmental characteristics were included in the model as features to demonstrate sample use of the method. All descriptions are from the UK Biobank Showcase, and no outliers were removed. Individuals that answered ”prefer not to answer” or ”do now know” to any given question were treated as missing values. All features are taken from the baseline assessment, the same point in time when the BMI phenotype was measured. The following environmental and personal features collected at baseline were evaluated:

Description	Data field
Age when attended assessment centre	21003
Genetic sex	22001
Number of days/week walked 10+ minutes	864
Minutes spent walking per day	874
Number of days/week of moderate physical activity 10+ minutes	884
Duration of moderate activity per day	894
Number of days/week of vigorous physical activity 10+ minutes	904
Duration of vigorous activity per day	914
Alcohol intake frequency	1558
Sleep duration	1160
Processed meat intake	1349
Beef intake	1369
Lamb/mutton intake	1379
Pork intake	1389
Cheese intake	1408
Milk type used	1418
Illness, injury, bereavement, stress in last 2 years	6145

2.1 Age when attended assessment centre

Age at the initial assessment visit (2006-2010) during which participants were recruited and provided consent.

2.2 Genetic sex

Sex as determined from genotyping analysis.

2.3 Physical activity

To measure the degree of physical activity, the duration of walking, moderate activity and vigorous activity per day were added with equal weight. The duration of any given activity per day is set to zero if an individual spent no days during the week with more than 10 minutes of that activity.

2.4 Alcohol intake

Participants were asked how frequently they consumed alcohol, with potential responses never, only on special occasions, one to three times a month, one to three times a week, three or four times a week, or daily or nearly daily.

2.5 Sleep duration

Participants were asked to report how many hours of sleep they got in a 24 hour period.

2.6 Saturated fat intake

Participants were asked how frequently they consumed each food item, from never to daily. Frequency of beef, lamb, mutton, pork, cheese and milk intake per week was added with equal weight.

2.7 Stressful events

We treated this as a binary variable, such that those that have not experienced any of the categories listed in the "Illness, injury, bereavement, stress in last 2 years" variable during the past two years are represented by the value zero, and the rest were set to one.

2.8 Treatment of categorical features and correlation plot

XGBoost does not automatically take into account categorical features. Sex, alcohol consumption and sleep duration can be considered categorical features, but as sex is a binary feature, while alcohol consumption and sleep duration are ordinal features, a split between two categories for these features in a regression tree is meaningful, and therefore the features can be treated as they are. The correlation of the final seven environmental features were investigated further by computing the Pearson's correlation between all pairs of features by excluding missing values. No pair of features showed Pearson's correlation r larger than 0.2, and we therefore treat these features as if they were independent of each other when computing the SHAP values. Correlations between environmental features and SNPs are also surprisingly not very small. Even though there exist dependence between SNPs and environmental features, the effects are so small that we also in this case regard them to be independent to each other when computing the SHAP values.

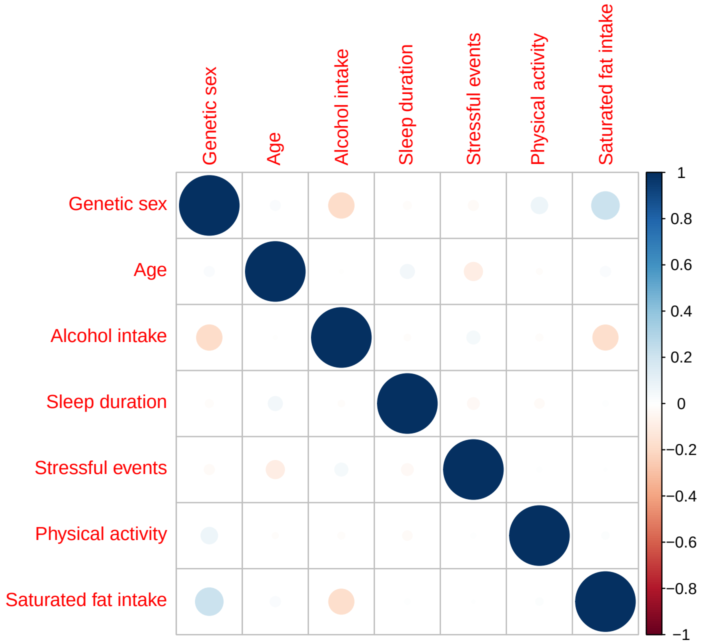

Figure 1: Pearson's correlation, r, between environmental features.

3 The minimum number of random subsets to choose in the ranking process

In Phase 1 of the method described in the main article, we perform a ranking process for the SNPs using a combination of random subsets of SNPs with cross-validation. Here we show the probability calculations guiding the choice of the number of random subsets of SNPs that we use, first for one SNP and then for a SNP pair.

3.1 Number of subsets for single SNP sampling

We have a total of R SNPs, and draw $S < R$ SNPs without replacement. Let $A = 1$ denote the case where we study one randomly sampled subset of S SNPs, and $A = a$ the case where we study a different samples. The question is how large a at least should be in order to investigate the whole genome to a sufficient extent.

Let C_j be the number of times a particular SNP j is chosen among all $A = a$ subsets. Since the SNPs are randomly sampled without replacement, the probability that SNP j is contained in at least one of the a subsets, $P(C_j \geq 1|A = a)$, is given by:

$$P(C_j \geq 1|A = a) = 1 - P(C_j = 0|A = a) = 1 - P(C_j = 0|A = 1)^a = 1 - \big(1 - \frac{S}{R}\big)^a,$$

since $P(C_j = 0|A = 1)$ is given from the corresponding hypergeometric distribution:

$$P(C_j = 0|A = 1) = \frac{\binom{1}{0}\binom{R-1}{S}}{\binom{R}{S}} = 1 - \frac{S}{R}.$$

If we want the probability to be larger than some preferred value p, we get the inequality referred to in the main article:

$$a \geq \frac{\log(1-p)}{\log(1-\frac{S}{R})}. \tag{1}$$

However, after the SNPs are randomly sampled, we also perform a pruning to minimize the correlation in the sample as explained in Section 4, so the number of subsets to create should be even larger than this.

3.2 Number of subsets for pair SNP sampling

Similarly, assume the SNPs to be randomly sampled, and let $C_{j,k}$ be the number of times SNP j and SNP k are present simultaneously in a total of a subsets. We then have:

$$\begin{aligned} P(C_{j,k} \geq 1|A = a) &= 1 - P(C_{j,k} = 0|A = a) = 1 - P(C_{j,k} = 0|A = 1)^a \\ &= 1 - \big(1 - P(C_{j,k} = 1|A = 1)\big)^a \\ &= 1 - \left(1 - \frac{S(S-1)}{R(R-1)}\right)^a, \end{aligned}$$

since $P(C_{j,k} = 1|A = 1)$ is given from a corresponding hypergeometric distribution:

$$P(C_{jk} = 1|A = 1) = \frac{\binom{2}{2}\binom{R-2}{S-2}}{\binom{R}{S}} = \frac{S(S-1)}{R(R-1)}.$$

For this probability to be larger than a preferred value p, we get the inequality referred to in the main article:

$$a \geq \frac{\log(1-p)}{\log\left(\frac{S(S-1)}{R(R-1}\right)}. \tag{2}$$

Again, the total number of subsets should be larger due to the need for SNP pruning to ensure low correlation among the SNPs. Anyhow, inequalities (1) and (2) can be used as guidance as to how many subsets should at least be created.

4 SNP pruning with PLINK1.9

When creating the subsets explained in Section 3.1 (the ranking process) of the main article, we create a subset of S SNPs with mutually low correlation together with G randomly sampled individuals. This is implemented by using both R and PLINK1.9 [4].

First, S^* SNPs and G individuals are sampled with equal probability and without replacement. Next we apply the PLINK1.9 function −−`indep-pairwise` with the following parameter values window size = 50 kb, step size = 5kb and $r^2 = 0.2$ in order to get a subset of S SNPs were all pairs of SNPs within a region of 50 kilobases have squared Pearson's correlation less than 0.2. SNPs that are more than 50 kilobases from each other are not expected to correlate to any significant extent. Pearson correlation measures linear dependency, and therefore zero correlation does not imply independence in general. We will anyhow rely on r^2 as a measure of independence due to its fast computation on large amounts of data. In the example analysis we manually find, by trial and error, the appropriate size of S^* corresponding to the chosen value for S.

In a similar manner, the PLINK1.9 function −−`indep-pairwise` can be used to obtain a subset of SNPs with mutually low correlation based on some ranked set of SNPs, as in Section 3.2 (model fitting process) in the main article. However, the ranked list of SNPs should be added as a .frq-datafile via −−read-freq, where the column variable MAF is edited such that it does not denote the minor allele frequencies, but some feature importance score of each SNP. The larger the score is, the higher priority the SNP will have to be kept among the subset.

5 Running BOLT-LMM on the ranking data

In the obesity example, we run BOLT-LMM on the ranking data (from Phase 1) with obesity as trait in order to rank the importance of each SNP based on the their computed p-values by using the BOLT-LMM-infinitesimal mixed-model statistic [2]. BOLT-LMM is intentionally constructed for quantitative traits and not for case-control traits such as obesity, but it can be applied by treating the binary trait as a quantitative trait. The caveat is however that the p-values may be invalid. However, the p-values computed have been shown to be valid as long as the MAFs of each SNP are larger than 1%, and that the case fraction is larger than 30% for a sample of 50 000 individuals [2]. The ranking data has a case fraction of 43 %, MAF greater than 1 % and 80 000 individuals, and so we regard the p-values computed as valid. Obesity and features were defined as described in Appendix B in the main article. Categorical features in the model were genetic sex, alcohol intake frequency, sleep duration (in hours), and any events of illness, injury, bereavement, or stress in the previous two years. Quantitative features were physical activity, saturated fat

intake, and age at initial assessment. All features excluding genetic sex were self-reported during the initial assessment.

6 Computations of SHAP values

The SHAP value, $\phi_{i,j}(\mathbf{x}_i)$, for a model $f(\mathbf{x}_i)$, individual i and feature j given all features $\mathbf{x}_i$ is defined in Lundberg et al. [3] and Janzing, Minorics, and Blöbaum [1] as:

$$\phi_{i,j}(\mathbf{x}_i) = \sum_{\mathcal{S}\subseteq\mathcal{M}\setminus\{j\}} \frac{|\mathcal{S}|!(M-|\mathcal{S}|-1)!}{M!} \Big[E[f(\mathbf{X}_{i,\mathcal{S}\cup\{j\}} = \mathbf{x}^*_{i,\mathcal{S}\cup\{j\}}, \mathbf{X}_{i,\overline{\mathcal{S}\cup\{j\}}})] - E[f(\mathbf{X}_{i,\mathcal{S}} = \mathbf{x}^*_{i,\mathcal{S}}, \mathbf{X}_{i,\overline{\mathcal{S}}})] \Big] \tag{3}$$

where $E[f(\mathbf{X}_{i,\mathcal{S}\cup\{j\}} = \mathbf{x}^*_{i,\mathcal{S}\cup\{j\}}, \mathbf{X}_{i,\overline{\mathcal{S}\cup\{j\}}})]$ is the expected prediction when only the values of the feature subset $\mathcal{S}$ as well as feature j, denoted $\mathbf{x}^*_{i,\mathcal{S}\cup\{j\}}$, are known, while the vector of unknown values from the complement set, $\mathbf{X}_{i,\overline{\mathcal{S}\cup\{j\}}}$ are regarded as a random vector. Notice that $S \cup \overline{S} = \mathcal{M}$.

6.1 SHAP values for tree ensemble models

We consider a tree ensemble model where the prediction, $f(\mathbf{x}_i)$, is a linear sum of outputs from all regression trees given features $\mathbf{x}_i$. By the linearity property of expectation, the marginal expectation, $E[f(\mathbf{X}_{i,\mathcal{S}} = \mathbf{x}^*_{i,\mathcal{S}}, \mathbf{X}_{i,\overline{\mathcal{S}}})]$, given in Equation (3) is equal to the sum of the marginal expectation of the output from each regression tree, denoted $E[f_\tau(\mathbf{X}_{i,\mathcal{S}} = \mathbf{x}^*_{i,\mathcal{S}}, \mathbf{X}_{i,\overline{\mathcal{S}}})]$:

$$E[f(\mathbf{X}_{i,\mathcal{S}} = \mathbf{x}^*_{i,\mathcal{S}}, \mathbf{X}_{i,\overline{\mathcal{S}}})] = \sum_{\tau=1}^{T} E[f_\tau(\mathbf{X}_{i,\mathcal{S}} = \mathbf{x}^*_{i,\mathcal{S}}, \mathbf{X}_{i,\overline{\mathcal{S}}})].$$

The marginal expectation for each regression tree, assuming only continuous features, is mathematically expressed as:

$$E[f_\tau(\mathbf{X}_{i,\mathcal{S}} = \mathbf{x}^*_{i,\mathcal{S}}, \mathbf{X}_{i,\overline{\mathcal{S}}})] = \int_{\mathbf{x}_{i,\overline{\mathcal{S}}}} f_\tau(\mathbf{X}_{i,\mathcal{S}} = \mathbf{x}^*_{i,\mathcal{S}}, \mathbf{X}_{i,\overline{\mathcal{S}}} = \mathbf{x}^*_{i,\overline{\mathcal{S}}}) p(\mathbf{X}_{i,\bar{\mathcal{S}}} = \mathbf{x}^*_{i,\overline{\mathcal{S}}}) d\mathbf{x}_{i,\overline{\mathcal{S}}}, \tag{4}$$

where we denote $\mathbf{x}^*_i = (\mathbf{x}^*_{i,\mathcal{S}}, \mathbf{x}^*_{i,\bar{\mathcal{S}}})$ as the constant vector where all feature values are known. As each regression tree f_τ only takes a distinct number of values equal to the number of leaves B_τ in the regression tree, the integral in (4) can be expressed as a sum of integrals:

$$E[f_\tau(\mathbf{X}_{i,\mathcal{S}} = \mathbf{x}^*_{i,\mathcal{S}}, \mathbf{X}_{i,\overline{\mathcal{S}}})] = \sum_{k=1}^{B_\tau} c_{\tau,k} \int_{\mathbf{x}_{i,\overline{\mathcal{S}_{\tau,k}}}} p(\mathbf{X}_{i,\overline{\mathcal{S}}} = \mathbf{x}^*_{i,\overline{\mathcal{S}_{\tau,k}}}) d\mathbf{x}_{i,\overline{\mathcal{S}_{\tau,k}}},$$

where each $\mathbf{x}^*_{i,\overline{\mathcal{S}_{\tau,k}}}$ is such that $f_\tau(\mathbf{x}^*_i = (\mathbf{x}^*_{i,\mathcal{S}}, \mathbf{x}^*_{i,\overline{\mathcal{S}_{\tau,k}}})) = c_{\tau,k}$ where $c_{\tau,k}$ is leaf value number k for tree τ.

If we assume the complement subset $\bar{\mathcal{S}}$ of features are mutually independent, the integral can be further partitioned into a product of integrals , where each integral will be integrated over the range of the corresponding feature in $\bar{\mathcal{S}}$ that leads to the path from root to leaf node with leaf node value $c_{\tau k}$:

$$E[f_\tau(\mathbf{X}_{i,\mathcal{S}} = \mathbf{x}^*_{i,\mathcal{S}}, \mathbf{X}_{i,\overline{\mathcal{S}}})] = \sum_{k=1}^{B_\tau} c_{\tau,k} \prod_{\ell=1}^{l} \int_{x_{i,\ell}=a_{\ell,\tau,k}}^{b_{\ell,\tau,k}} p(X_{i,\ell} = x^*_{i,\ell}) dx_{i,\ell},$$

where $x_{i,\ell}$ denotes the feature value of feature number ℓ among a total of l unknown features in the subset $\bar{\mathcal{S}}$, while $(a_{\ell,\tau,k}, b_{\ell,\tau,k})$ is the range in which feature number ℓ must be integrated over in order to get the output value $c_{\tau,k}$ for regression tree τ. For features in $\bar{\mathcal{S}}$ that are not present in the regression tree τ, these features can take any value. We define the value of the corresponding integrals in the product operator to be one.

What remains in order to compute the marginal expectation given in Equation (3) is to estimate each of the integrals given above. In Lundberg et al. [3] these are estimated by using the proportion of samples in each node in each tree in the training phase of the tree ensemble model that goes in the same direction from a particular node to another. Under the assumption of mutual independence this is a reasonable estimate, but the estimate naturally relies on the total number of individuals that are used for estimation, and so these estimations will be poorer the deeper the trees are. Finally, and most importantly, in order to compute the SHAP values for a tree ensemble model, Lundberg et al. [3] have constructed an algorithm with polynomial running time, $O(TLD^2)$, for maximum depth D and leaves L.

7 Logistic regression with different additivity assumptions

In the main article, all likelihood ratio tests are based on the assumption of both additive marginal effects and additive interaction effects. Here we provide two additional tests with less stricter additive assumptions.

For the case of SNP-SNP interactions, the first model is unconstrained in both main effects and interactions [5]:

$$\begin{aligned}&\text{logit}(P(Y_i = 1|g_{i,a}, g_{i,b}, \mathbf{x}_{i,c})) = \\&\mathbf{x}_{i,c}^T\gamma + \alpha_1 I(g_{i,a} = 1) + \alpha_2 I(g_{i,a} = 2) + \beta_1 I(g_{i,b} = 1) + \beta_2 I(g_{i,b} = 2) \\&+ \nu_{11} I(g_{i,a} = 1) I(g_{i,b} = 1) + \nu_{12} I(g_{i,a} = 1) I(g_{i,b} = 2) \\&+ \nu_{21} I(g_{i,a} = 2) I(g_{i,b} = 1) + \nu_{22} I(g_{i,a} = 2) I(g_{i,b} = 2),\end{aligned} \tag{5}$$

where $\mathbf{x}_{i,c}^T$ is a vector of features such as intercept, age, environmental features and principal components, γ is the vector of corresponding parameters for each feature, $I()$ is the indicator function, α_1, α_2, β_1 and β_2 are marginal effects of the SNPs $g_{i,a}$ and $g_{i,b}$ when the genotype value is one or two respectively, while ν_{11}, ν_{12}, ν_{21} and ν_{22} are unconstrained interaction parameters for $g_{i,a}$ and $g_{i,b}$.

When testing the presence of interaction effects, the null hypothesis is $\nu_{11} = \nu_{12} = \nu_{21} = \nu_{22} = 0$, with null model:

$$\begin{aligned}&\text{logit}_{H_0}(P(Y_i = 1|g_{i,a}, g_{i,b}, \mathbf{x}_{i,c})) = \\&\mathbf{x}_{i,c}^T\gamma + \alpha_1 I(g_{i,a} = 1) + \alpha_2 I(g_{i,a} = 2) + \beta_1 I(g_{i,b} = 1) + \beta_2 I(g_{i,b} = 2).\end{aligned} \tag{6}$$

If we assume additive interaction effects, corresponding to $\nu_{11} = \nu$, $\nu_{12} = \nu_{21} = 2\nu$ and $\nu_{22} = 4\nu$, we get the alternative model:

$$\begin{aligned}&\text{logit}(P(Y_i = 1|g_{i,a}, g_{i,b}, \mathbf{x}_{i,c})) = \mathbf{x}_{i,c}^T\gamma + \alpha_1 I(g_{i,a} = 1) + \alpha_2 I(g_{i,a} = 2) \\&+ \beta_1 I(g_{i,b} = 1) + \beta_2 I(g_{i,b} = 2) + \nu g_{i,a} g_{i,b}.\end{aligned} \tag{7}$$

We will then have two new tests based on the following null and alternative models: Models (6) and (5) in the case of no assumptions and models (6) and (7) in the case of additive interactions. We denote these tests as Test 1 and Test 2 respectively. The test applied in the main article is denoted as Test 3 with null and alternative models:

$$\text{logit}_{H_0,add}(P(Y_i = 1|g_{i,a}, g_{i,b}, \mathbf{x}_{i,c})) = \mathbf{x}_{i,c}^T\gamma + \alpha g_{i,a} + \beta g_{i,b}. \tag{8}$$

$$\text{logit}_{H_1,add}(P(Y_i = 1|g_{i,a}, g_{i,b}, \mathbf{x}_{i,c})) = \mathbf{x}_{i,c}^T\gamma + \alpha g_{i,a} + \beta g_{i,b} + \nu g_{i,a} g_{i,b}. \tag{9}$$

For the case of SNP-environment interactions, the logistic models will look similar in the case where the environmental feature is discrete. For the case where the environmental feature, $x_{i,e}$, is continuous, the unconstrained Test 1 will for instance have the following alternative model:

$$\begin{aligned}\text{logit}(P(Y_i = 1|g_{i,a}, x_{i,e}, \mathbf{x}_{i,c})) = \mathbf{x}_{i,c}^T\gamma + \alpha_1 I(g_{i,a} = 1) + \alpha_2 I(g_{i,a} = 2) + \beta_e x_{i,e} \\+ \phi_1 I(g_{i,a} = 1) x_{i,e} + \phi_2 I(g_{i,a} = 2) x_{i,e},\end{aligned} \tag{10}$$

where β_e, ϕ_1 and ϕ_2 are the marginal effect of the environmental feature, and interaction effects respectively.

The results when applying all three tests for each of the interactions based on both the evaluation data and all individuals is given in Table 1.

Table 1: Results from all likelihood ratio tests with different assumptions of additivity. The tests are applied on the top four ranked interactions found from the model explainability process based on the evaluation data.

**Test**	**Interaction**	***p*-value LRT**
Test 1 evaluation data	rs171329 and rs180743	0.49
Test 1 all individuals	rs171329 and rs180743	0.0063
Test 2 evaluation data	rs171329 and rs180743	0.85
Test 2 all individuals	rs171329 and rs180743	0.024
Test 3 evaluation data	rs171329 and rs180743	0.85
Test 3 all individuals	rs171329 and rs180743	0.024
Test 1 evaluation data	rs17817449 and genetic sex	0.96
Test 1 all individuals	rs17817449 and genetic sex	0.00022
Test 2 evaluation data	rs17817449 and genetic sex	0.79
Test 2 all individuals	rs17817449 and genetic sex	4.78e-05
Test 3 evaluation data	rs17817449 and genetic sex	0.77
Test 3 all individuals	rs17817449 and genetic sex	4.09e-05
Test 1 evaluation data	rs17817449 and saturated fat intake	0.59
Test 1 all individuals	rs17817449 and saturated fat intake	0.0019
Test 2 evaluation data	rs17817449 and saturated fat intake	0.45
Test 2 all individuals	rs17817449 and saturated fat intake	0.0017
Test 3 evaluation data	rs17817449 and saturated fat intake	0.44
Test 3 all individuals	rs17817449 and saturated fat intake	0.0017
Test 1 evaluation data	rs757318 and rs12123815	0.48
Test 1 all individuals	rs757318 and rs12123815	0.49
Test 2 evaluation data	rs757318 and rs12123815	0.25
Test 2 all individuals	rs757318 and rs12123815	0.71
Test 3 evaluation data	rs757318 and rs12123815	0.25
Test 3 all individuals	rs757318 and rs12123815	0.71

Even though the three statistical tests have different assumptions, the p-values for the three tests for each interaction do not vary greatly. Therefore, in this case, the assumptions of additivity do not have any significant impact of the computed p-values.

8 PCA plots - Evaluation data and full dataset

Figure 2: PCA plot for the first and second principal components for unrelated individuals in the full dataset.

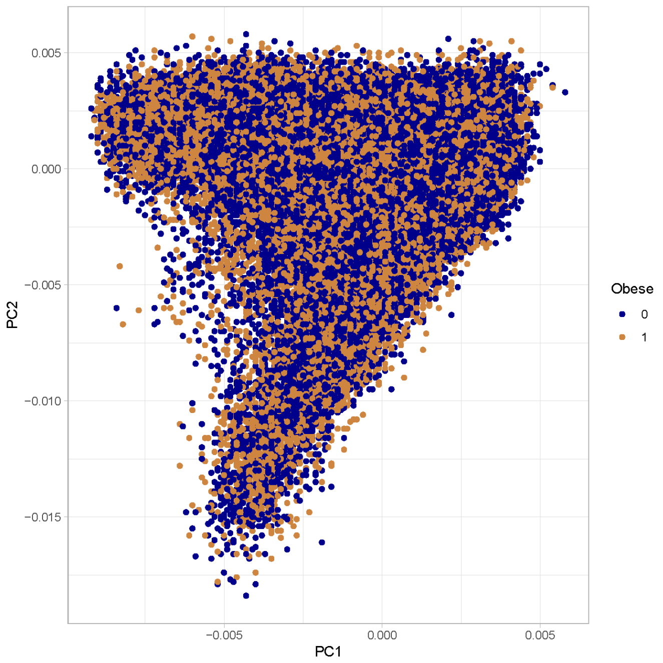

Figure 3: PCA plot for third and fourth principal components for unrelated individuals in the full dataset.

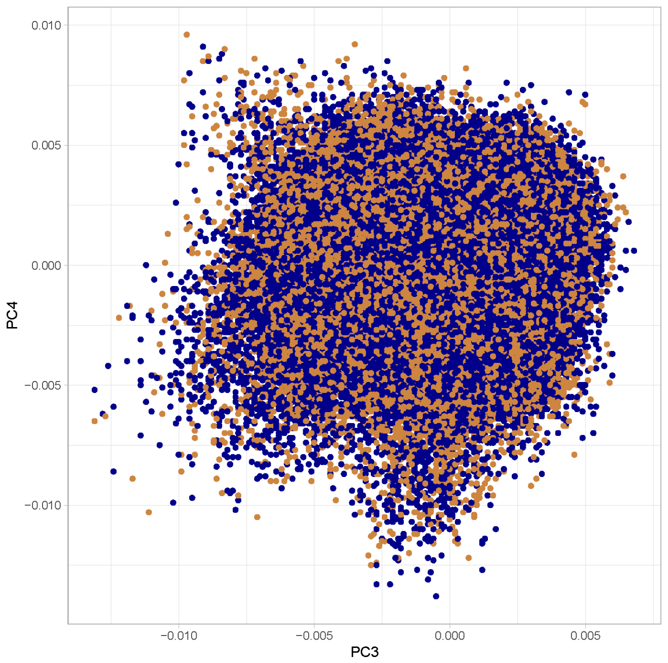

Figure 4: PCA plot for first and second principal components for unrelated individuals in the evaluation dataset.

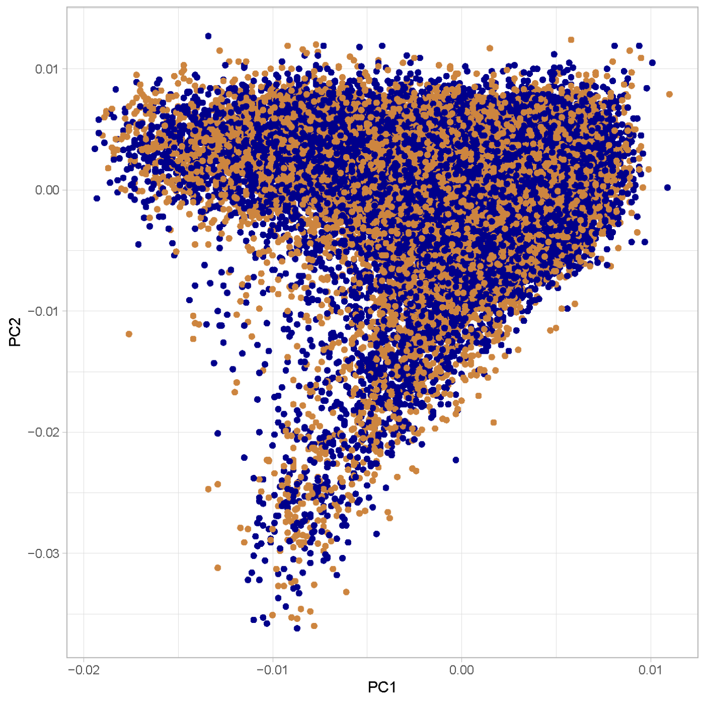

Figure 5: PCA plot for third and fourth principal components for unrelated individuals in the evaluation dataset.

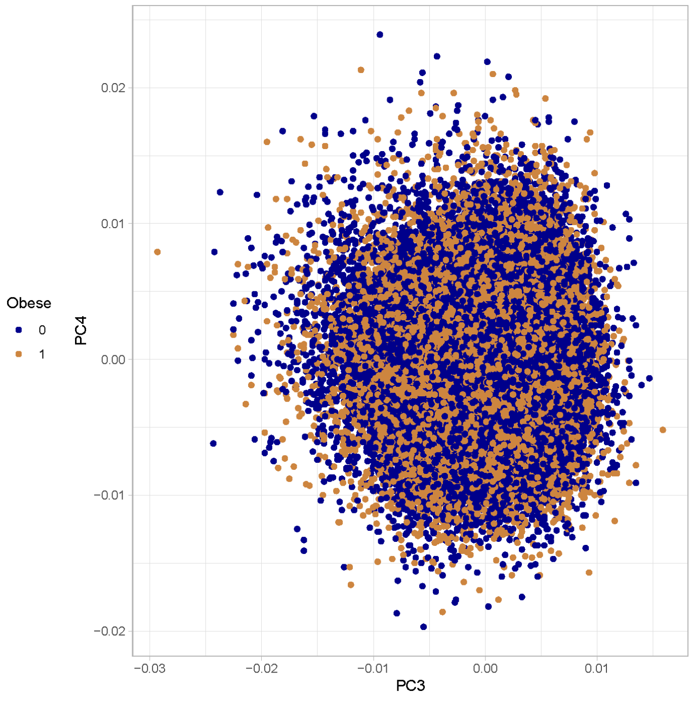

References

[1] Dominik Janzing, Lenon Minorics, and Patrick Blöbaum. "Feature relevance quantification in explainable AI: A causal problem". In: *arXiv:1910.13413 [cs, stat]* (2019).

[2] Po-Ru Loh et al. "Mixed-model association for biobank-scale datasets". In: *Nature Genetics* 50 (July 2018), pp. 906–908.

[3] Scott M. Lundberg et al. "From local explanations to global understanding with explainable AI for trees". In: *Nature Machine Intelligence* 2.1 (Jan. 2020), pp. 56–67.

[4] Shaun Purcell et al. "PLINK: A Tool Set for Whole-Genome Association and Population-Based Linkage Analyses". In: *American Journal of Human Genetics* 81.3 (2007), pp. 559–575.

[5] Zhaoxia Yu, Michael Demetriou, and Daniel L. Gillen. "Genome-Wide Analysis of Gene-Gene and Gene-Environment Interactions Using Closed-Form Wald Tests". In: *Genetic Epidemiology* 39.6 (2015).
